# Detecting application layer DDoS attack using an advanced signature detection algorithm

**DOI:** 10.1038/s41598-026-56617-z

**Published:** 2026-06-11

**Authors:** Abdul Ghafar Jaafar, Md Asri Ngadi, Nazri Kama, Nazhatul Hafizah Kamarudin, Khairol Shapawi

**Affiliations:** 1 Faculty of Artificial Intelligence, Universiti Teknologi Malaysia, Kuala Lumpur, Malaysia; 2 Faculty of Computing, Universiti Teknologi Malaysia, Johor Bahru, Johor Malaysia; 3 Centre for Cyber Security, Faculty of Information Science and Technology, Universiti Kebangsaan Malaysia, Bangi, 43600 Selangor Malaysia; 4 KYROL Security Labs Sdn. Bhd, Cyberjaya, Malaysia

**Keywords:** Engineering, Mathematics and computing

## Abstract

Application-layer Distributed Denial of Service (App-DDoS) attacks are an ongoing issue in the cyber security world. The attack constructs request headers and uses a large number of channels to disrupt targeted services, such as an automated attack tool. A variety of approaches have been attempted, but the detection of attacks through the identification of forged request headers is a significant gap in the research. Signature detection, which shows a strong ability to accurately identify attacks with low false positive and false negative rates, can be used to address this challenge. The paper introduces a new detection method to categorize traffic as malicious or legitimate by analyzing the request headers of the traffic. To address the concerns of various researchers regarding outdated attack patterns and the lack of datasets available for public research, the dataset used in this research is recent and representative of real-world App DDoS attack patterns. The signature detection demonstrates promising performance in detecting the attack with the latest data set, which has strong implications for adaptation to real-world applications. The key contributions of this study are the proposed detection algorithms that can detect forged request headers at the initial stage, prior to their processing by the web server, and the practical analysis, which showed that the attack strategy approach relies on the manipulation of request headers. The hybrid feature selection method employed in this research was proven to be workable and successfully identified the features which contribute significantly to the detection performance with an accuracy of 96.93%, a precision of 99.11%, a recall of 97.55%, and an F1-score of 98.32%. These results demonstrate that the signature-based detection is effective and appropriate for detecting the attack. Although Machine Learning (ML) is gaining traction, signature-based detection can still be effective for checking signatories generated by the attack in the request headers.

**Supplementary Information:**

The online version contains supplementary material available at 10.1038/s41598-026-56617-z.

## Introduction

Application-layer Distributed Denial of Service (App-DDoS) attacks are a type of malicious traffic sent to the target server, designed to overwhelm the server while appearing to be legitimate requests; frequently, botnets are used in the attack. The goal of this attack is to deny users access to content on the Internet and make services less available^1–4^. A high number of HTTP requests (GET/POST) can be sent to the server that hosts the online resource to exploit it. As a result, they become unable to provide services and quality declines as the server becomes overloaded with incoming traffic that isn’t needed^5^. This disruption results in financial losses due to service continuity issues and a lack of users’ trust. Moreover, an enormous rise in HTTP-based attacks is observed. For instance, in 2024, Kaspersky detected almost 57,116 DDoS attacks. This represents a 67% surge from earlier periods^6^. HTTP/HTTPS DDoS attacks grew 487% from 2019 to 2022^7^; netscout,^8^, and in Q4 2023, Cloudflare recorded the biggest DDoS attack it has ever mitigated, reaching around 201 million Requests per Second (RPS). This scenario points to the rise of hyper-volumetric DDoS attacks that impose heavy loads on Internet infrastructure^9,10^. An HTTP DDoS attack can be particularly hazardous as it can be directed at a variety of industries. However, according to Kaur et al.^11^, the cryptocurrency industry was the most targeted industry for HTTP DDoS attacks, followed by the gaming and gambling industries in Q4 2024. The attack is just another example of the ongoing and changing nature of HTTP DDoS attacks against online services.

## Problem background

The detection of DDoS attacks in apps cannot simply be done based on server traffic volume because a large number of concurrent users could generate a high volume. In this case, traffic volume alone can cause false positive and false negative readings. No inspection is done to verify the authenticity of the request header, and the presence of HTTP Strict Transport Security (HSTS) is not enough to verify the authenticity of the request header at the server level. HSTS ensures secure connections by redirecting all HTTP requests to HTTPS^12,13^. As a result, this allows hackers to spoof headers, making it difficult to identify malicious traffic among legitimate traffic. This problem has been partially addressed by Mohammadi et al.^14^, who proposed a solution to detect App DDoS attacks by measuring the number of headers generated by the source requester. Nevertheless, incomplete headers are not the only patterns produced by the attack. Praseed and Thilagam^15^ highlighted that the attack is strikingly similar to legitimate traffic and remains difficult to detect.

Aside from that, the request headers also include a query string that hackers can manipulate to generate forged traffic to overwhelm target servers. The forged query string is generated from an ASCII string because, in automated attack tools, the ASCII encoding maps each character to a unique numeric value. Even though several methods are available, such as Strict-Transport-Security and Content-Security-Policy, none are designed to secure request headers^16^. By exploiting properties of the ASCII encoding, attackers generate forged query strings that closely resemble those of legitimate users.

Although websites increasingly deploy HTTPS (HTTP over TLS), this security channel primarily encrypts the transport channel. It does not eliminate HTTP request headers such as user-agent, referrer, host, request URI, query string, accept, encoding, and connection. Furthermore, modern web infrastructure terminates TLS at reverse proxies or load balancers before traffic reaches the web server. The HTTP protocol is widely significant in computing due to its diverse applications, including IoT, cloud, edge computing, and live video streaming, and its functionality that supports flexibility, compatibility, and RESTful interactions^17,18^. The protocol is, however, a weak one and needs extra security measures. While several techniques have been used, there is still a gap in research regarding the detection of forged request headers generated by the attack. The adverse effect of this problem is that a web server must deal with both legitimate and illegitimate traffic at the same time. This causes the web server to run out of resources because of the high workload, and eventually, can result in a loss of service or failure of the system.

## Contribution

This research proposes algorithms to detect App DDoS attacks by inspecting request headers. To the best of the author’s knowledge, previous studies have not comprehensively applied the request header feature in DDoS detection. Therefore, prior studies have had difficulty detecting recent, realistic attack patterns generated by the attack. The attack manipulates the request header to appear real. Thus, to systematically understand the technique, a combination of three approaches is necessary: static and dynamic code analysis, and statistical analysis. Hence, this research introduces a detection strategy for App DDoS attacks based on the inspection of request header features. The goal is to ensure early detection of the attack before the web server becomes overwhelmed. The main contributions of the paper are as follows.

- The architecture for developing a custom App DDoS dataset in a controlled experimental environment to address the research gap caused by the limited availability of recent App DDoS datasets.
- Systematic feature selection process that integrates static code analysis, dynamic code analysis, and statistical analysis. This process identifies precise request-header features to improve detection performance.
- The detection algorithms for App DDoS attack. The formulation decomposes the detection process into three modular designs: forged User-Agent detection, forged Referrer detection, and request URI query-string detection.
- Practical analysis of automated attack-tool behavior reveals how App DDoS tools manipulate request headers to make attack traffic appear similar to legitimate traffic.

## Problem analysis

This section analyzes automated attack tools used by hackers to compromise web servers, employing both static and dynamic analysis, unlike previous studies that often rely solely on datasets to analyze attack patterns. The analysis begins by studying the code structure without executing it. Consequently, the code is executed in a safe environment, and incoming traffic is observed and analyzed using Wireshark to understand the attack pattern. Static and dynamic analyses provide a comprehensive understanding of the attack’s characteristics and facilitate the systematic identification of the strategies attackers use to overwhelm the web server.

### False user-agent and referrer

**Fig. 1:**
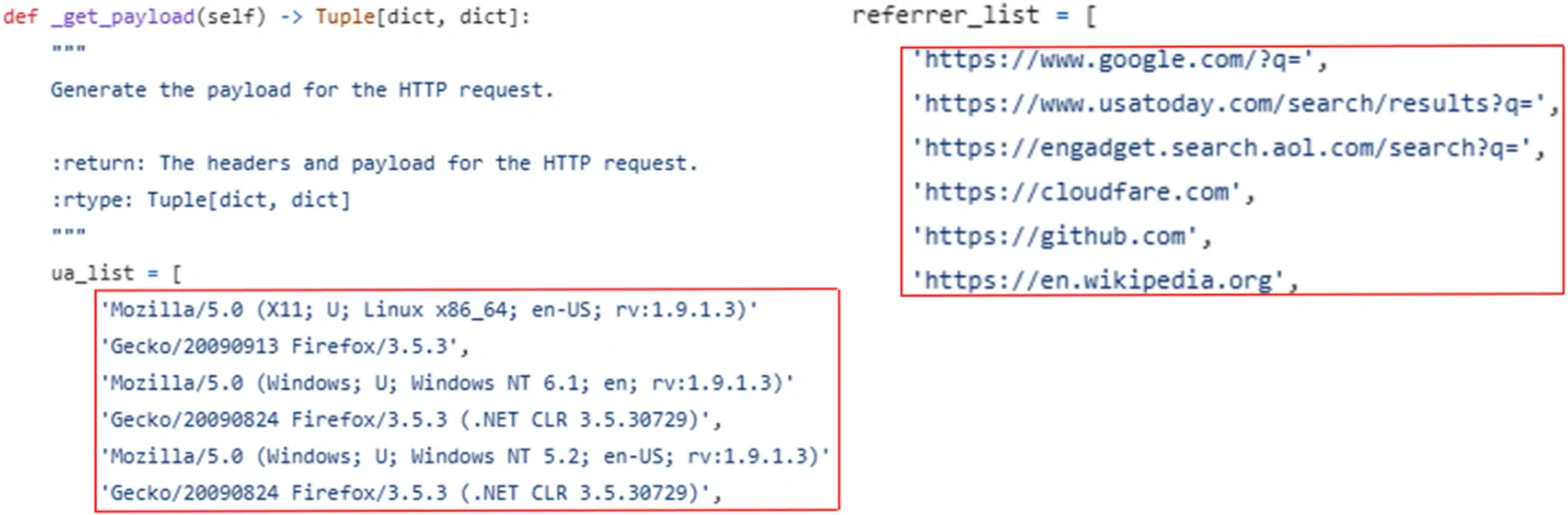
False user-agent and referrer.

An app DDoS attack generates substantial amounts of forged user-agent and referrer headers to evade detection. For instance, Hulk Version 3 uses the *_get_payload* function, which contains a container named *ua_list* that lists authentic user-agent strings. The tools use randomized techniques. Thus, the user-agents appear to come from various browsers and are authentic. The tools also use valid URLs within the container *referrer_list*. The URL appears valid because it uses legitimate domains such as Google, GitHub, and Wikipedia. These URLs are randomly selected during the execution of the App DDoS attack to emulate legitimate web traffic. Through this mechanism, the incoming internet traffic to the web server appears to originate from several sources. Consequently, the web server will be overwhelmed with forged traffic that appears authentic. This problem has been partially addressed by Mohammadi et al.^14^, who proposed a solution to detect HTTP DDoS attacks by measuring the number of headers generated by the source requester. Nevertheless, incomplete headers are not the only patterns produced by the attack. Praseed and Thilagam^15^, stated that the attack is very similar to legitimate traffic and is not easily detectable. The code for forging user agents and referrers is shown in Fig. 1.

### Request queries manipulation

**Fig. 2:**
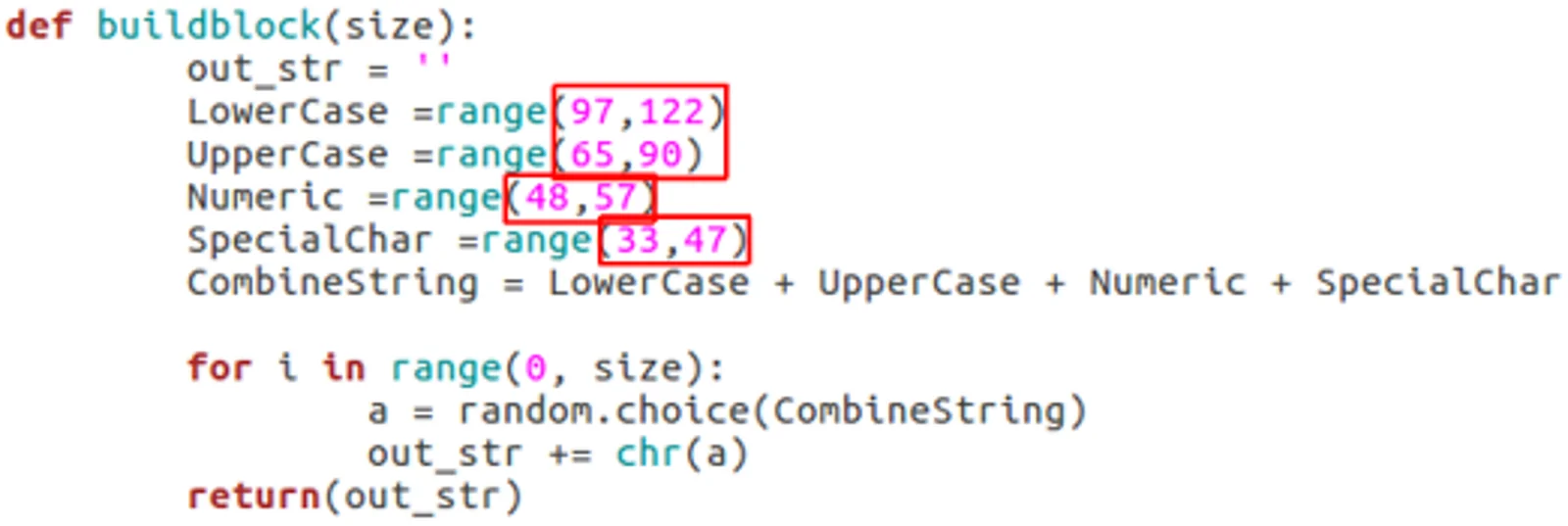
Malicious automated tools code with ASCII characters.

The App DDoS attack also used forged request queries that hackers manipulated to generate large volumes of requests that could overwhelm a server. Hackers employ ASCII characters to enhance the App DDoS attacks’ ability to mimic legitimate user requests. This situation becomes significantly complex because the ASCII characters can also be used with automated attack tools to construct queries, repeatedly combining lowercase and uppercase letters, numbers, and special characters. The advanced techniques used by hackers involve the use of valid, semantically meaningful strings that appear legitimate to human readers. Even with a rate limit in place, the technique has a downside when dealing with DDoS attacks because it cannot provide early detection or alerting, and the number of concurrent connections must be correctly configured. A too-low threshold can lead to false positives, while a higher threshold can lead to false negatives. Such mechanisms are usually based on certain thresholds, which attackers can easily play with to evade detection^19^. If thresholds are set too high, some attack traffic may pass unnoticed. However, if set too low, normal traffic may be misclassified as malicious^20^. Table 1 demonstrates the ASCII table, while Fig. 2 illustrates the use of the ASCII code in automated attack tools that generate false request queries.

**Table 1:** ASCII character.

Type	ASCII character	Symbol generate
Lower case	97 and 122	a-z
Upper case	65 and 90	A-Z
Numeric	48 and 57	0–9
Special character	33 and 57	!“#$%&`()*+’-./

### Abusing the server accessibility

**Fig. 3:**
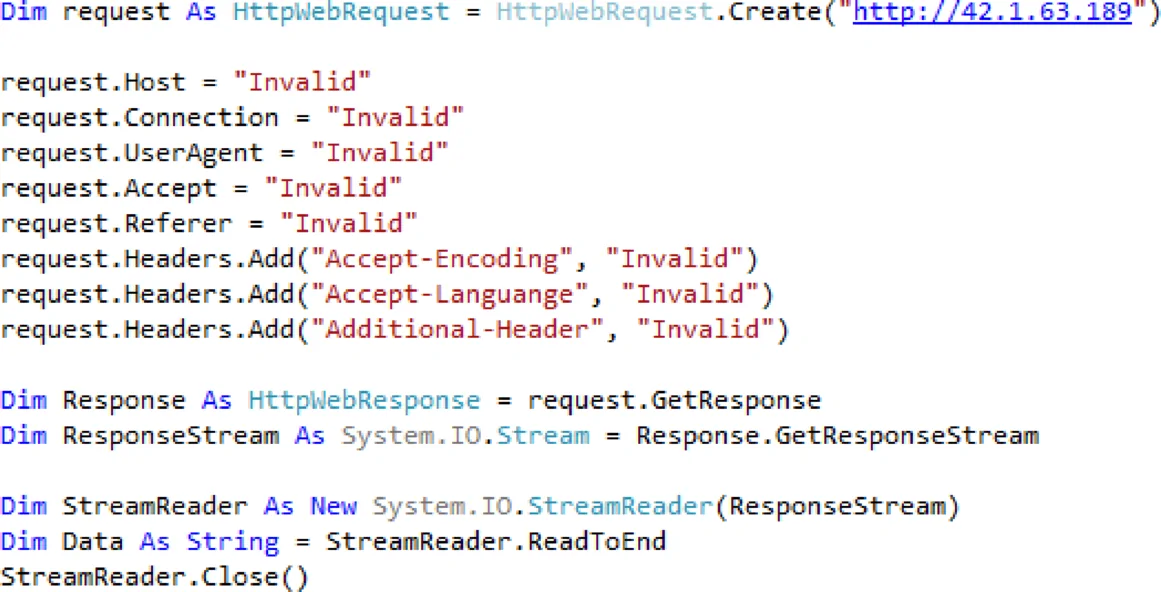
Dummy header.

**Fig. 4:**
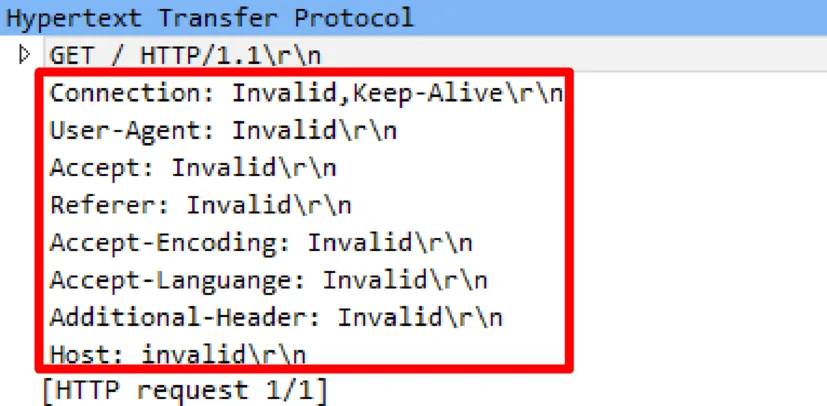
Wireshark filtering for dummy header.

The platforms users use to access servers also require further attention, since connections to a web server can be established in various ways, not just through web browsers but also via Telnet, Secure Shell (SSH), and custom tools. For example, BitTorrent clients may use HTTP to connect to websites and download files, and laboratory digitization systems may use HTTP for data transfer within network infrastructures^21^. Although several methods are available, such as Strict-Transport-Security and Content-Security-Policy, they are not designed to verify the legitimacy of headers^16^. Based on the experiment conducted, no filtering of HTTP request headers (such as user-agent, referrer, connection, language, or accept-encoding) was observed at the web server level. As a consequence, hackers may create dummy headers that appear legitimate, thereby misleading security analysts during inspection and allowing malicious requests to evade application-layer filtering mechanisms. Figure 3 demonstrates the header components, and Fig. 4 highlights the result. This result proof that the headers can be easily constructed and altered, which allows attackers to exploit application-layer vulnerabilities.

**Fig. 5:**
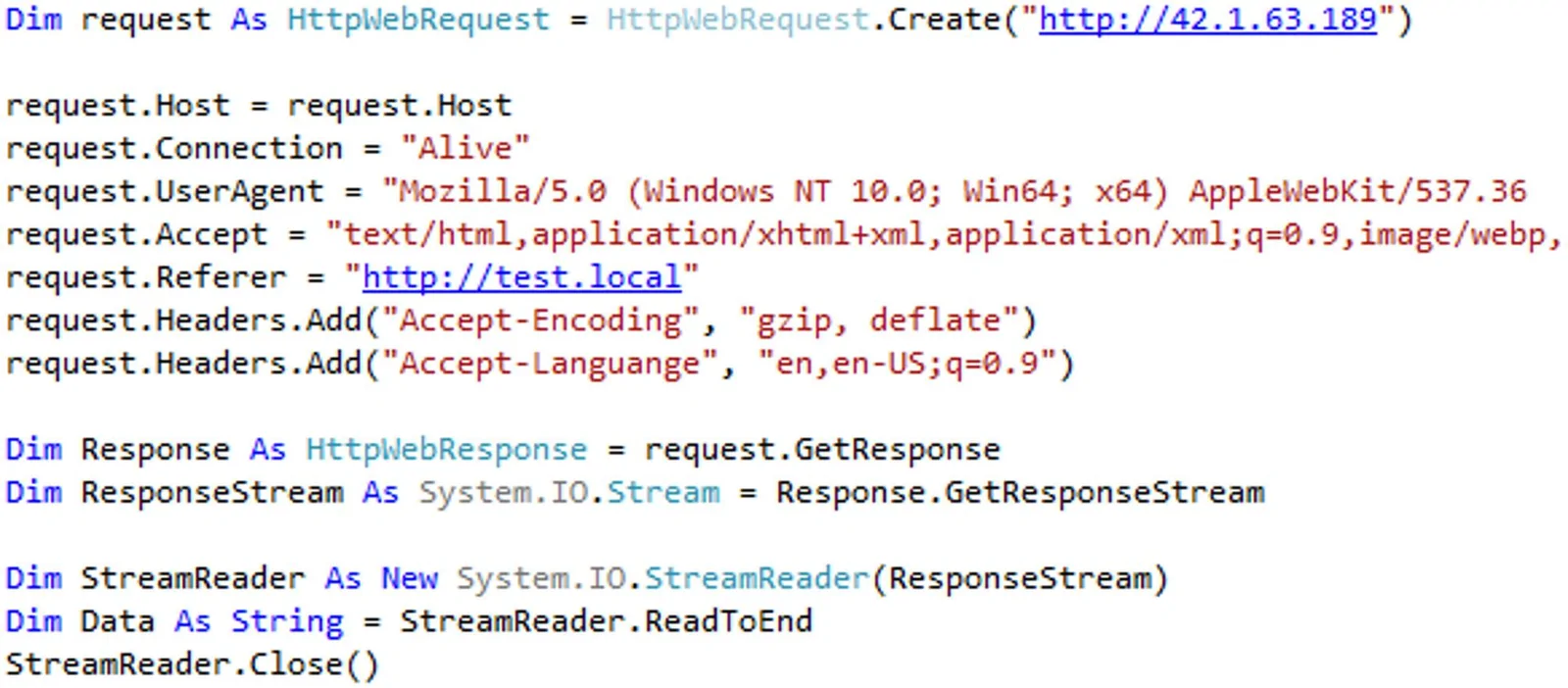
Header code.

**Fig. 6:**
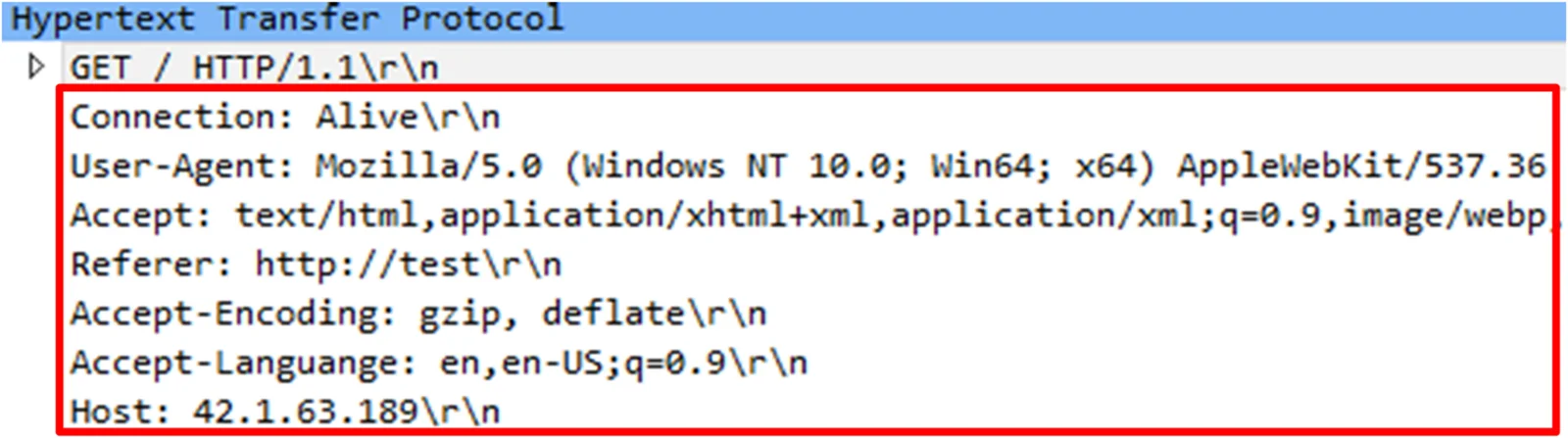
Header result.

The problem analysis continues to evaluate the susceptibility of HTTP headers to abuse. The results indicate that the header is easily exploitable and that the web server accepts the connection, as exhibited in Fig. 5, which includes code demonstrating the exploit. In contrast, the result is indicated in Fig. 6.

The analysis conducted in Sects.  4.1–4.3 revealed that automated attack tools manipulate request headers. However, the most persistent are user-agent, referrer, and request-query. Therefore, this will constitute the primary mechanism for mimicking legitimate client behavior and bypassing application-layer detection. Further analysis illustrated that the header can be easily manipulated, given that there is no strict authentication check. The experimental results of this section can be used as preliminary evidence that the header is an effective attribute for attack detection. Thus, these three headers are selected as they are directly observed in the manipulation patterns and can be explored and used in this research.

## Related work

App DDoS detection can be classified into signature and anomaly detection. By employing the signature-based approach, the system is capable of detecting the presence of illegitimate traffic and allows legitimate users to access web content with high detection rates, precision and accuracy. Signature-based algorithms have been reported to have high detection rates with low false-positive rates by Maniriho et al.,^22^. Aside from that, it can also identify both hackers and authentic users^23^, which is useful for distinguishing between users and hackers who use public proxies to access web content or execute HTTP DDoS attacks. Besides that, Jmila and Khedher ,^24^ observed that various machine-learning algorithms can be used for anomaly detection, including Naive Bayes, decision trees, support vector machines, and random forests. Nonetheless, according to Schmitt^25^, there is no consensus regarding the most effective machine-learning algorithm. Both types of detection have specific advantages and drawbacks.

### Related work based on signature-based detection algorithm

The signature-based algorithm is based on network behavior known as misuse, knowledge-based, rule-based, and pattern-based detection^26^. It relies on knowledge-based databases and predefined policies. Several studies adopted signature-based detection for HTTP DDoS attacks, owing to its ability to detect them immediately. Praseed and Thilagam^15^ utilized HTTP request headers to build an Early Detection Module (EDM) to detect DDoS attacks. The proposed work monitors incoming requests to ensure the source connection is not repeatable and compares traffic against the signature database to determine its status. Another approach to detecting the attack is to use redirection to identify the hacker’s identity, as suggested by Gonçalves et al.^27^. The detection can redirect HTTP requests to other locations to determine the authenticity of the source request. Hence, the study uses HTTP 302 redirects to differentiate incoming traffic from humans and automated tools and requires the user to click a link to confirm the source.

Mohammadi et al.^14^ proposed SHFD, a detection mechanism implemented as a defense module in Software-Defined Networking (SDN) to detect HTTP DDoS. SHFD extracts the source IP address from the SYN packet and adds it to the *HostReqList.* The incoming traffic will be detected as suspicious if the source IP address delivers incomplete request headers during a time window. Park et al.^28^ also deployed SDN to detect HTTP DDoS, categorized as flooding attacks (SDN). The scholar uses a web server to inspect incoming traffic and forward it to the SDN controller, blocking the connection if the incoming IP address is suspicious.

Another way to detect the attack is to use page separation and resource allocation strategies introduced by Patidar and Somani^29^. It has an allowlist mechanism that treats requests originating from this source as authentic traffic from this section, using an authentication channel once credentials are provided. Rao Varre and Bayana^30^ suggested using a request rate monitor to control the volume of incoming traffic and to analyze authentic and malicious traffic. Every request rate is monitored based on the server capacity allocated to each request. The traffic monitoring and rate-limiting concept is applied by Rao Varre and Bayana^30^ and Zhao et al.^31^, who introduced the concept of behavioral utility to model network patterns. The study posits that changes in network status caused by attacks can be characterized as malicious behavior patterns. The studies use load balancing and rate limits to control the maximum number of connections across different attack scales, and the exact mechanisms determine the threshold status.

### Related work based on an AI machine learning detection algorithm

In this context, Artificial Intelligence (AI) and Machine Learning (ML) have become significant tools in cybersecurity, especially for the identification of HTTP DDoS attacks. AI and ML provide advanced capabilities, including the ability to analyze vast amounts of network data, detect patterns, and differentiate between normal and abnormal behavior. The AI utilizes ML models, which include the classic models like Naive Bayes and decision trees, and the advanced ones like random forest, gradient boosting and deep learning, as mentioned by Sarker et al.^32^. As highlighted by Al-gethami and Aljuhani^33^, several ML models can be applied to detect HTTP DDoS attacks, such as K-Nearest Neighbors (KNN) and Random Forest. These models are frequently used to analyze network traffic patterns and identify anomalies, such as a sudden surge in HTTP requests that may indicate an attack. Another study by Alghazzawi et al.^34^ identified that combining multiple ML models, such as Convolutional Neural Networks (CNNs) with Bidirectional Long Short-Term Memory (BILSTMs), can effectively improve detection accuracy.

Several studies have proposed AI-based ML approaches to detect App DDoS attacks, such as Mohammadi et al.^35^, which adopt SDN and ML algorithms to detect and mitigate HTTP flooding attacks. The proposed detection is implemented in the SDN controller to monitor the HTTP connections and document each connection in the log table. All HTTP flows at the End of a time window are inspected to detect the presence of the attack.

Feng et al.^36^ suggested that a reinforcement-learning-based model can detect and mitigate HTTP DDoS. The module is designed to train multiple metrics related to server load, dynamic user behavior, and the victim network load. A similar detection matrix was proposed by Praseed and Thilagam^37^ using two-phase detection that leverages server logs and access traces to capture the behavioral dynamics of legitimate users in the form of annotated Probabilistic Timed Automata (PTA). The detection phase is designed to intercept and examine all incoming connections to detect traffic patterns that do not conform to the learned model. Another equivalent technique introduced by Abubakar et al.^38^ adopted traffic behavior analysis, packet header validation, protocol validation, and traffic matching with datasets.

Yu^39^ proposed a semi-supervised ML concept combining spectral clustering and random forest. The study utilized a dataset containing seven discrete feature variables: guest_login, logged_in, land_flag, is_host_login, service, and protocol_type. Beitollahi et al.^40^ also analyzed variables available in the dataset, which used a Genetic Algorithm (GA) for data collection, cleaning, normalization, and feature selection. The proposed detection distinguishes malicious requests based on features available in the dataset, such as protocol type, the percentage of connections with SYN errors, and the percentage of connections using the same or different services.

### Comparative analysis

Based on problem analysis conducted in Sect.  4 and the existing studies’ proposed work, there are several identified features and areas that are either lacking or have remaining issues, which are the motivating factors behind the present study, to improve App DDoS detection methods. The difficulty in detecting App DDoS attacks stems from the fact that the forged request headers generated by the attack are indistinguishable from legitimate ones. Moreover, the attack can generate massive traffic from a single machine using automated attack tools.

The attack can be addressed using signature-based detection because automated attack tools often generate repeatable forged-header patterns. Even though some attack patterns are indistinguishable, this research argues that, with an appropriate detection strategy, they can be effectively detected. Therefore, Table 2 compares existing signature-based DDoS detection and discovers that prior studies mainly focus on traffic volume, flow behavior, URL repetition, request rate, HTTP redirection, page semantics, CAPTCHA responses, and incomplete header transmission. These methods provide useful detection strategies for network-layer flooding, HTTP flooding, slow-rate attacks, and resource-consumption attacks. However, they do not specifically address forged and repeatable request-header behavior produced by automated App DDoS tools. The proposed work fills this gap by focusing on user-agent, referrer, and request-URI query-string manipulation through request-header inspection before web-server processing or after TLS termination.

**Table 2:** Comparison table.

Author	Feature scope	Detection point	Assumptions	Attack coverage
Myint Oo et al.,^26^	Volumetric and asymmetric flow statistics, average number of flow packets, flow bytes, their variations, and traffic duration	SDN application layer	Flooding attacks are distributed normally, and attack traffic fundamentally differs from normal traffic in volume and symmetry	Flooding-based DDoS attacks - UDP flooding and TCP SYN flooding
Praseed and Thilagam,^15^	HTTP request URL patterns Periodic repetition Similarity in the request sequence	Server-side or edge via continuously monitoring incoming streaming data windows.	Attackers utilize automated tools or botnets that follow pre-defined scripts without randomization, producing repetitive and Predictable URL sequences	Non-randomized Application Layer DDoS attacks, including single/multiple URL floods and asymmetric attacks.
Park et al.,^28^	HTTP request packet rates (packets per unit time) and semantic processing capabilities via CAPTCHA test responses.	threshold monitoring at the targeted web server, active intervention, and CAPTCHA challenges.	Legitimate clients can identify complex image patterns to solve CAPTCHA, whereas botnets lack this semantic capability and cannot properly respond	Heavy-traffic HTTP DDoS flooding attacks.
Gonçalves et al.,^27^	HTTP 302 status code and the Location header	Server on the edge of the Local System, coordinating with a Storer module and an SDN switch.	Malicious botnets use lightweight malware that does not fully implement the HTTP protocol, and they are unable to follow simple HTTP redirection challenges	Botnet-based HTTP flood Distributed Denial-of-Service attacks.
Patidar and Somani,^29^	Requested page semantics and concurrent TCP connections per source IP	On a Docker container master node, managed by HAProxy to route requests and monitor concurrent connection thresholds	Different web pages create inherently different levels of resource contention, and authenticated users are legitimate and should be prioritized	Application-layer DDoS attacks aimed at fraudulent resource consumption.
Mohammadi et al.^14^	Incomplete HTTP headers mapped to specific source IPs over dynamic time windows	Evaluating flow statistics gathered from OpenFlow edge switches	Malicious hosts alter normal traffic uniformity, and attackers behave distinctly from flash crowds	Slow-rate HTTP flooding and high-rate HTTP flooding attacks.
Propose work	User-agent, referrer, and request URI query string	Request-header inspection before web-server processing or after TLS termination.	Automated App DDoS tools generate forged but repeatable header patterns that can be detected through rule-based inspection	Forged user-agent, referrer, and irrelevant or manipulated query-string patterns.

## Dataset validity and constraint

There is a significant challenge in obtaining the recent App DDoS dataset, which is one of the primary concerns because the existing datasets, such as Darpa98-99, KDD99, CAIDA, MIT Lincoln, and FIFA, are considered obsolete and do not adequately reflect current network traffic patterns^41,7^. Many of the most popular datasets are not web-specific, and the attacks included are constrained and outdated, as highlighted in Díaz-Verdejo et al.,^42^. Shafi et al.,^43^ have analysed the 16 publicly available datasets in detail. They noted 15 gaps that make it difficult to develop effective detection methods, such as real-time data and incomplete feature sets. However, some of the existing DDoS datasets are not available for public use due to security reasons, which limits researchers’ access to up-to-date data^44^. Moreover, Vashishtha and Chatterjee ,^45^ also discussed the importance of datasets to accurately represent the current traffic patterns and various attack scenarios. Numerous studies have extensively covered issues related to datasets in cybersecurity, including DDoS, and their details can be found here^41,46,1,47^.

Kenyon et al.,^47^ elaborated that the absence of adequate representation and inconsistent quality in intrusion detection datasets can significantly undermine the effectiveness of detection algorithms when trained on low-quality data. Zhang,^48^ also agreed that data variations can substantially affect the outcomes of the detection process. Kenyon et al.,^47^ noted a shortage of standardized metrics for measuring dataset quality. Consequently, researchers typically have to rely on several factors, for instance, the author’s reputation, the quality of published results, and personal experience, to assess the quality of a dataset. A recent survey by Maniriho et al.,^22^ highlighted that the dataset lacks valuable details. Therefore, this research chose to collect the dataset through the execution of real automated attack tools, as further explained in the Methodology Section.

## Methodology

**Fig. 7:**
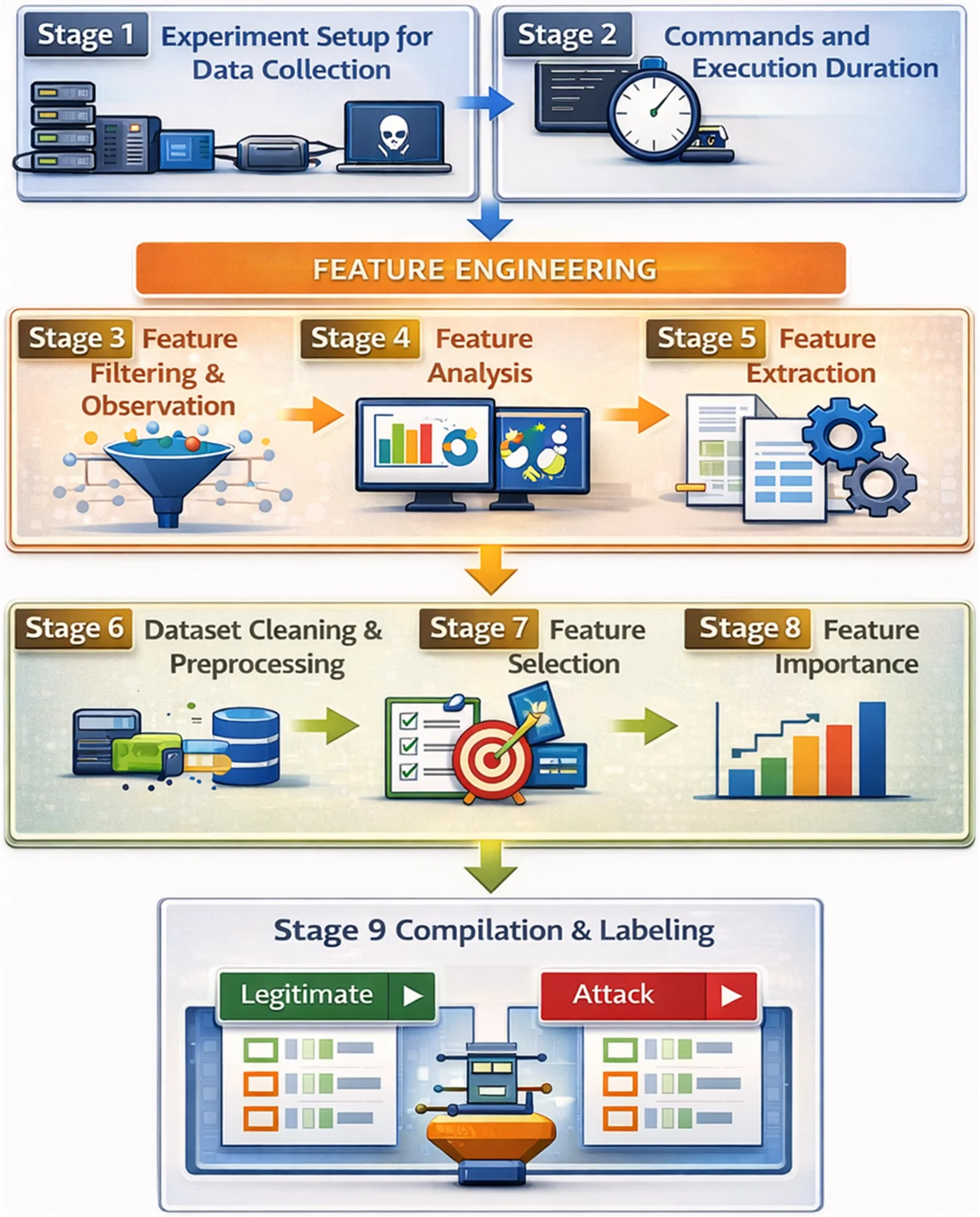
Methodology framework.

The methodology used in this research consists of nine steps, beginning with an experimental setup for data collection using a real device to collect an actual real-world dataset. Command and execution are the processes in which automated attack tools are executed. This stage continues with feature filtering and observation to isolate specific data and examine its characteristics. The analysis extracts features from the collected data, while the feature extraction identifies valuable features based on the analysis. Consequently, data cleaning and preprocessing were initiated to remove redundant and duplicate values. The feature selection was conducted through code analysis. Subsequently, feature importance analysis was performed to identify the most significant features. Lastly, the data were compiled and labeled. The methodology framework is illustrated in Fig. 7 and explained in detail in Sects.  7.1 to 7.10.

### Experiment setup for data collection

**Fig. 8:**
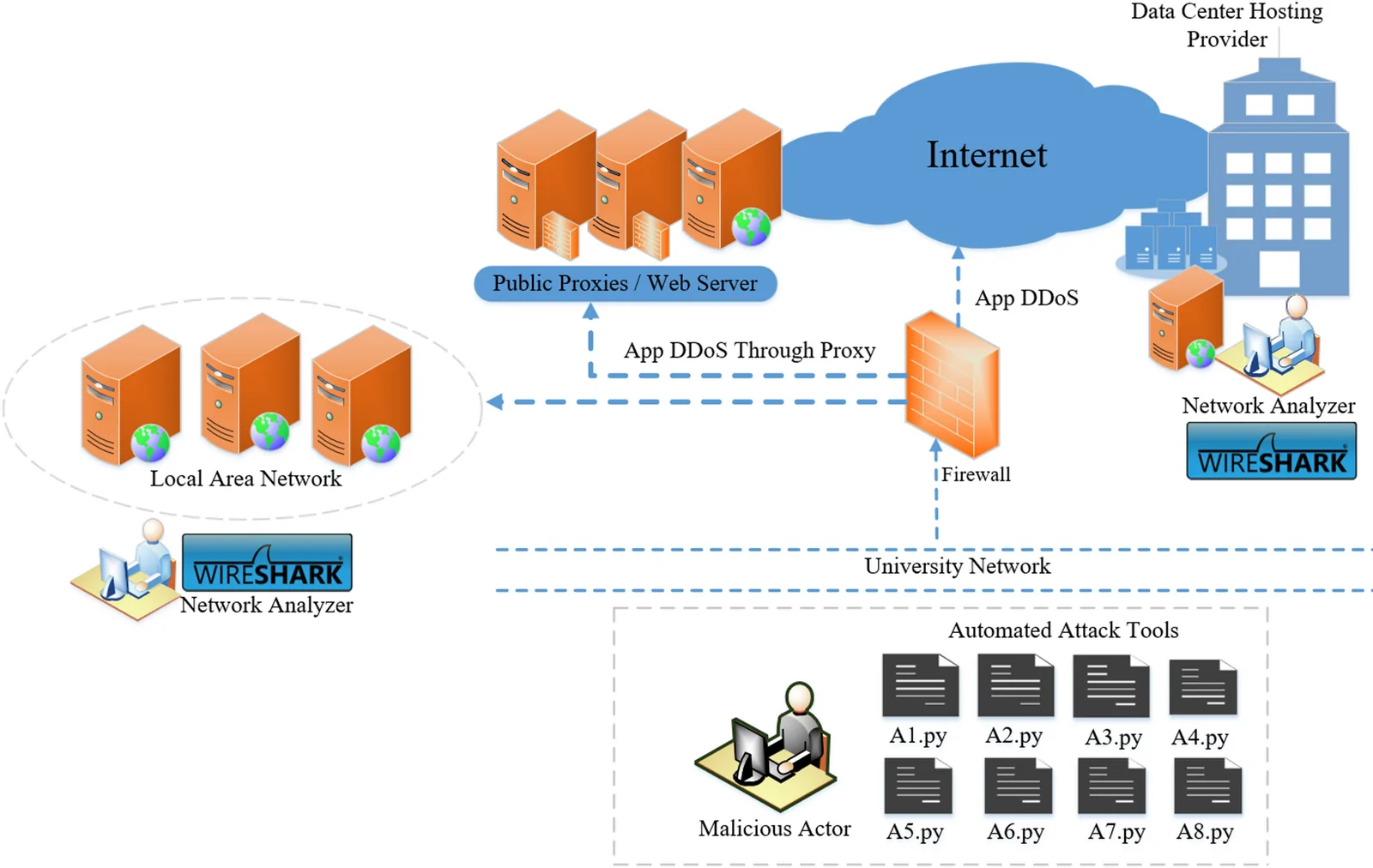
Experimental architecture.

The experiment is performed on a physical machine and a virtual machine, both running the Ubuntu operating system, that will be used to install and run automated attack tools, and a dedicated target machine running Microsoft Windows with an HTML web page installed within the Internet Information Services (IIS) server. Traffic patterns had to be captured using a traffic capture tool like Wireshark, and the Amaranten firewall had to be set up to mimic the production network architecture. This architecture tries to resolve the issue mentioned by Bahashwan et al.,^49^, that datasets are generated in virtual environments. However, the issues of privacy and control of the severity of the attacks may not reflect the diversity of network applications, sizes and traffic patterns of real-world networks. Chahal et al.,^7^ emphasized that many organizations are hesitant to share attack data from their real-world environments because of reputation, customer trust, media, security and legal liability concerns. Hence, the experimental architecture developed in this research can address the challenges highlighted by prior studies and enable effective detection. Figure 8 illustrates the experimental architecture.

The scope of this research is only to execute three automated attack tools, although eight App DDoS tools are illustrated in Fig. 8. The rationale for this limitation is to facilitate replication by other researchers in order to develop a customized, up-to-date App DDoS attack pattern. Through this approach, researchers can analyze the source code of automated attack tools and execute them in a safe environment to identify potential attack strategies adversaries may employ. If this happened, researchers would always stay ahead, since whatever tactic they use is already known, leading to better detection of the attack. Implementing this method also addresses the concern about the limitations of the existing dataset for App DDoS attacks, which is outdated and publicly available, as elaborated in Sect.  6.

### Commands and execution duration

Three datasets, known as ChiHulk, GoldenEye, and BlackHorizon, sourced from GitHub, are used in this research. The number of datasets used to collect the attack pattern is sufficient, as reliance on a single dataset may limit the detection of real-world scenarios^50^. The ChiHulk (A1_ChiHULK.py) runs for 10 min and generates a total of 1,284,394 packets. In contrast, GoldenEye, designated as A2_goldenEye.py, and BlackHorizon, designated as A3_BlackHorizon.py, were each run for 5 min, generating 299,260 and 303,327 packets, respectively. The attack duration is selected to balance capturing meaningful attack behavior with maintaining system stability and preventing resource overload, while minimizing disruption to the experimental setup. This approach allows for the collection of meaningful data without compromising the integrity of the system under test^51^. Execution of DDoS attacks for 5 to 10 min is sufficient to cause significant resource exhaustion, service disruption, and QoS degradation, while also providing a realistic window for detection and analysis of attack effects in experimental and operational environments^15,52,53,4^. The automated attack tools operate over the HTTP protocol to target the intended server. Although most servers use HTTPS to secure the transaction channel, it does not eliminate request headers, as encrypted traffic is decrypted prior to server-side processing. Table 3 summarizes the generation of attack traffic.

**Table 3:** Command and duration.

No.	Command	Duration	Total packets
1.	python A3_BlackHorizon.py http://42.1.63.189	5 min	303,327
2.	python A1_ChiHULK.py http://42.1.63.189	10 min	128,4394
3.	python A2_goldenEye.py http://42.1.63.189	5 min	299,260

### Feature filtering and observation

**Fig. 9:**
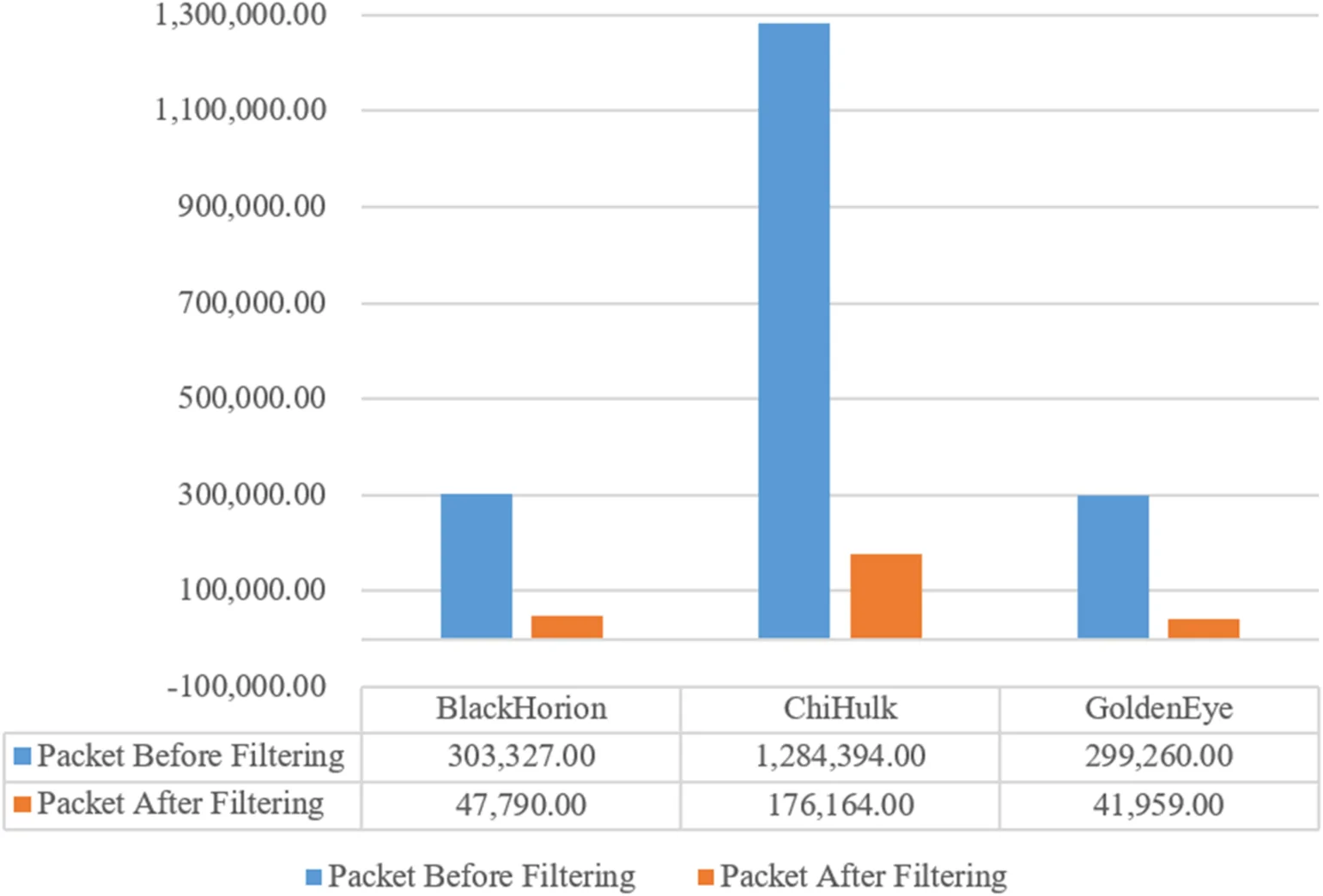
Traffic volume comparison.

Datasets produced in controlled environments often contain redundant or irrelevant features, which can hinder effective analysis. As a result, filtering is required to remove noise, select relevant features, and focus on specific protocols to ensure the dataset is suitable for analysis^54–60^. Only the application-layer features were kept, and irrelevant network traffic was filtered out. After discarding irrelevant features, 13 application headers were found; this means that more research is needed to identify which application headers are relevant for the detection of effective HTTP DDoS. In terms of attack packets, BlackHorizon generated 47,790, ChiHulk generated 176,164, and GoldenEye generated 41,959. These values reflect the varying intensity of and approaches to requesting in each of the tools used in the experiment. Figure 9 presents a graphical comparison of traffic volume by tool to facilitate more precise interpretation. At the same time, Table 4 demonstrates the attack’s application-layer features.

**Table 4:** Application layer headers.

No.	Name	Description
1.	Length	Size of HTTP request packet
2.	Accept-encoding	Supported content encoding formats
3.	Accept-charset	Supported character encoding
4.	Host	Target server domain or IP
5.	User-agent	Client application identifier
6.	Request line	HTTP method and request URI
7.	Connection	Connection management directive
8.	Cookie	Session or tracking information
9.	Referrer	Source URL of the request
10.	Request URI query parameter	Parameters appended to URI
11.	Cache-control	Catching behavior directive
12.	Connection type	Type of connection handling
13.	Content-type	Payload media type

### Feature analysis

The research primarily analyses application headers, as they are a highly prevalent request type used in App DDoS attacks. The header has various features as illustrated in Table 4. Thus, to guide this research in executing feature analysis, the Wireshark filtering command is used, as indicated in Table 5.

**Table 5:** Filtering commands.

No.	Commands	Details
1.	http	Present only the HTTP protocol
2.	http.request	Present only GET requests instead of HTTP POST
3.	http.user_agent	Filter the user agent used by the client
4.	http.referrer	To filter the HTTP referrer, use it to access web content
5.	http.request.uri.query	Filter query submitted against the web server to process
6.	http.connection	Show the connection status between the client and the web server
7.	http.request && http	Show only GET requests traffic and the HTTP protocol

**Fig. 10:**
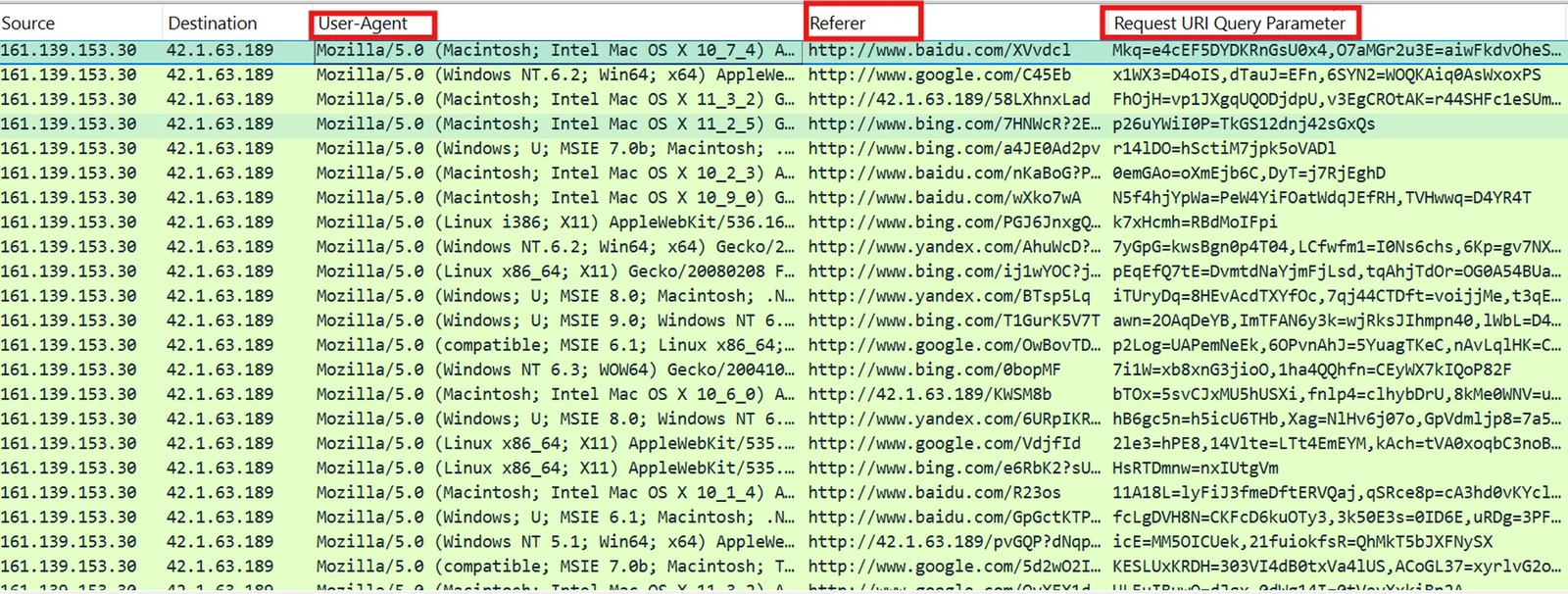
False user-agent, referrer, and request query.

The results of the feature analysis show significant findings: packet captures obtained with Wireshark reveal that application headers contain falsified user-agent, referrer, and query-string values. A variation of the user agent has been created, which contradicts the authentic version. A similar pattern is clearly observed, as search engines nowadays use HTTPS rather than HTTP. Aside from that, the query string is clearly invalid because it contains an unreadable string that contradicts the authentic version. The feature analysis results are consistent with the strategies used in the automated attack tools’ source code, as described in Sect.  4, indicating that these features have a major impact on the identification of App DDoS attacks. Figure 10 depicts traffic capture for user-agent, referrer, and request query, which provides supporting evidence for the above statement.

### Feature extraction–dataset transformation PCAP to CSV

Wireshark supports saving packet captures in Comma-Separated Values (CSV) format. Thus, feature extraction can be done by saving the PCAP files into CSV format. As PCAP stores each packet as a binary record, a transformation was necessary to extract relevant, human-readable fields while omitting low-level network details. Only application headers, such as referrer, query string, user-agent, accept, encoding, source, destination, connection, and host, were retained since the scope of this research is App DDoS attack detection. The Wireshark filtering command, as illustrated in Table 5, is reused to ensure that only the application-layer header is extracted. Other headers are excluded as they are not relevant to the scope of App DDoS attack, which heavily relies on the request header, as further explained in Sect.  4 of the problem analysis.

### Dataset cleaning and preprocessing

**Fig. 11:**
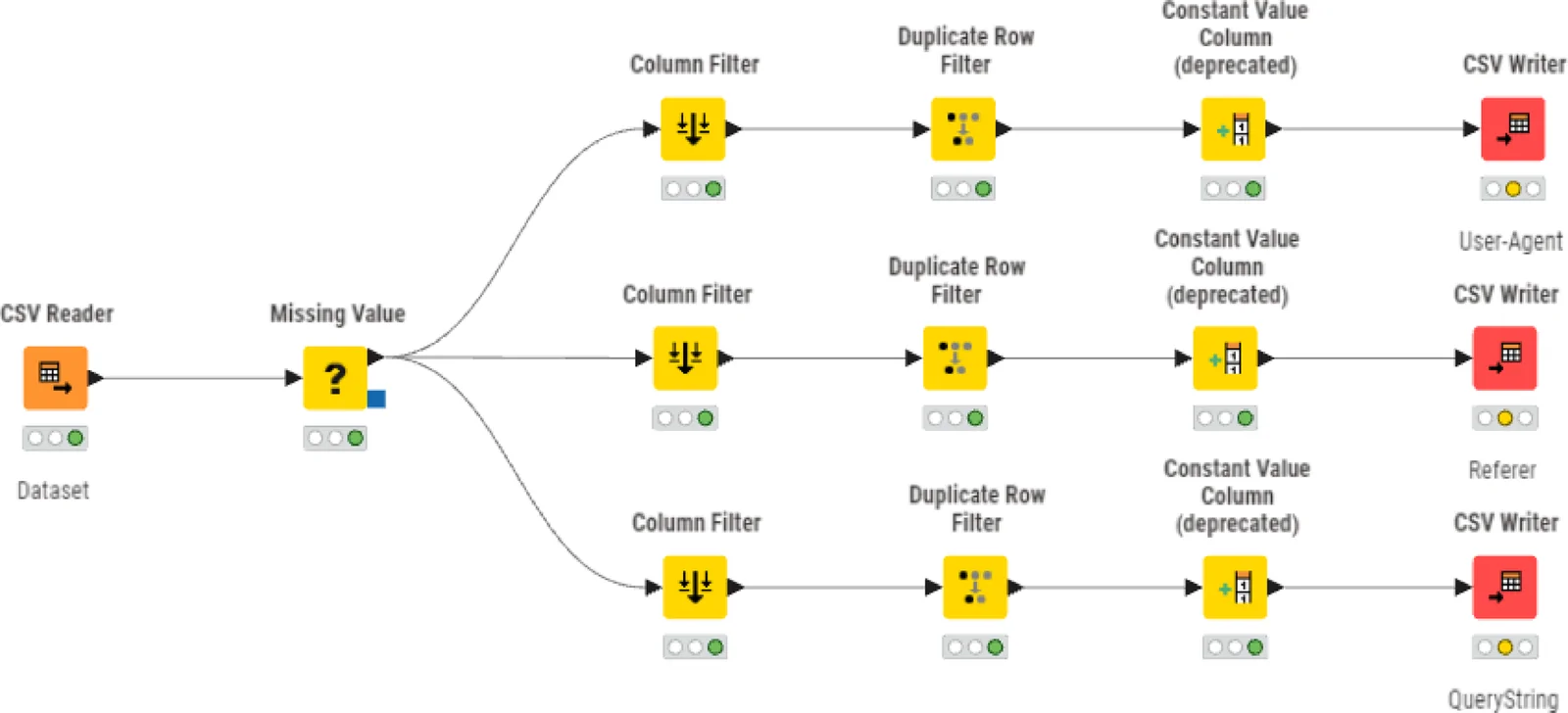
Process flow for dataset cleaning and preprocessing.

The data cleaning process was performed using the KNIME Analytics Platform to facilitate and accelerate data cleaning and preprocessing. In order to make this process run smoothly, there are some essential nodes: Missing Value, Column Filter, and Duplicate Row Filter. They are frequently applied and vital when automating cleaning tasks such as filling in missing data, deduping records, removing irrelevant attributes, and validating data formats. These steps are taken to make sure the final dataset is not inconsistent and redundant. After data cleaning and preprocessing, the results show that there is a reduction in the size of the user-agent, referrer and query string. The record is reduced following this process, as illustrated in Table 6, and the process flow is summarized in Fig. 11.

**Table 6:** Total attack dataset before and after cleaning.

Dataset name	User-agent		Referrer		Request query	
	Before cleaning	After cleaning	Before cleaning	After cleaning	Before cleaning	After cleaning
BlackHorizon	47,790	5,945	47,790	12,045	47,790	12,045
ChiHulk	176,164	344	176,164	35,136	176,164	44,189
GoldenEye	41,959	5,348	41,959	10,451	41,959	10,451

### Benign dataset collection

The data was divided into three sections: user-agent, referrer, and query string. The user-agent dataset is from GitHub, by the author Berlin^61^, and includes user agents for different web browsers, including Chrome, Firefox, Safari, and Opera. The dataset for legitimate referrers and/or query strings was synthetically generated due to the lack of authentic sets in public repositories and the outdatedness of datasets, as described in Sect. 6. Although the referrer URLs were synthetically created, they were carefully validated to ensure they were active and available. The real query strings are also checked for validity, and to make sure that they are actually query strings created by the user. Past studies also report on the use of synthetic data to simulate attack traffic for testing intrusion detection systems (IDS) enhanced with the use of honeypots^62,63^, and to increase the number of samples in a malware database with LLMs Jelodar et al.^64^. Many real attack scenarios cannot be captured reliably, and synthetic traffic is useful to get the exact ground-truth for attack/benign classification, as stated by Pinto et al.^65^. This means researchers have to generate them synthetically^66^.

**Table 7:** Total benign dataset.

Dataset name	Total record in dataset	Total record use for evaluation
User-agent	5999 (collect from GitHub)	400
Referrer	998 (synthesized)	998
Request query	1000 (synthesized)	1000

Table 7 illustrates the details of the benign dataset used for evaluation. The total number of records captured for the user-agent is 5999, and only 400 were used because they were sufficient to represent the main characteristics and diversity of legitimate user-agent patterns. The synthesized referrer dataset contains 998 records because two generated entries were excluded during validation due to invalid structures. In contrast, all 1000 synthesized request query records passed validation. The selected authentic benign records were sufficient because user-agent strings follow a predefined structure that includes several attributes, such as the browser name. Similarly, referrer and request query values follow valid URL and key–value parameter structures.

### Compilation and labeling

The forged and authentic user-agent, referrer, and query-string datasets were combined and labeled as suspicious and as an attack. The number of the clean dataset was considered reasonable and acceptable, as the problem analysis in Sect. 4 noted that the attack strategy rotates the headers. As a result, the attack will generate the same forged header with minimal differences. The total clean dataset, compiled with attack and benign indicators that are used for evaluation, is stated in Table 8.

**Table 8:** Dataset for evaluation.

Automated attack tools	User-agent	Referrer	Query string
BlackHorizon	6345 (Attack = 5945 + benign = 400)	13,043 (Attack = 12,045 + Benign = 998)	13,045 (Attack = 12,045 + Benign = 1000)
ChiHulk	744 (Attack = 344 + benign = 400)	36,134 (Attack = 35,136 + Benign = 998)	45,189 (Attack = 44,189 + Benign = 1000)
GoldenEye	5748 (Attack = 5348 + benign = 400)	11,449 (Attack = 10,451 + Benign = 998)	11,451 (Attack = 10,451 + Benign = 1000)

### Feature selection

The statistical method, which is typically used to identify feature correlations, has a downside: it cannot entirely explain the root causes of the attacks. Consequently, the accuracy of the attack detection process will be affected. Statistical approaches are practical for discovering feature correlations and their importance in attack detection for studies that do not investigate root causes^67–70^. Unlike the code analysis method, statistical approaches primarily rely on statistical relationships rather than directly examining malicious behavior patterns. This allows for the direct identification of features used by attackers, which can then be reused as detection features^71–74^. Therefore, feature selection in this research was conducted through code analysis. The results presented in Sect. 4, together with further analysis in Sect. 7.4, confirm that hackers use user-agent, referrer, and query-string headers to mimic real traffic patterns.

### Feature important

The feature analysis presented in Sect.  7.4 reveals that the user-agent, referrer, and query-string headers are the most critical indicators used by App DDoS. Nevertheless, to strengthen the selection of the headers, statistical analysis using a log-scaled diversity score was conducted. This decision to use this analysis is due to high-cardinality textual features such as user-agent, referrer, and query string, which often demonstrate extreme variability. The results indicate that the headers contribute a higher percentage, and this indicator shows that including user-agent, referrer, and query string can significantly improve detection performance. The result also explains the exclusion of other headers, as their use can affect detection performance. In addition, the results from Sect.  4 align with the feature importance results, showing that the user-agent, referrer, and query string receive higher scores than the other features, providing a strong indicator of their significant impact on attack detection. Tables 9, 10 and 11 show the result.

**Table 9:** Feature important for ChiHulk dataset.

Features name	Type	Feature importance
Query string	Text string	9.39327879
Referrer	Text string	9.191960544
User-agent	Text string	5.131707125
Source	IP string	0.608710754
Destination	IP string	0.608710754
Accept	Text string	0.608710754
Encoding	Text string	0.608710754
Connection	Text string	0.608710754
Host	Text string	0.608710754

**Table 10:** Feature important for BlackHorizon dataset.

Features name	Type	Feature importance
Referrer	Text string	10
Query string	Text string	10
User-agent	Text string	9.248640625
Accept	Text string	3.467355634
Encoding	Text string	3.467355634
Source	IP string	0.737666227
Destination	IP string	0.737666227
Connection	Text string	0.737666227
Host	Text string	0.737666227

**Table 11:** Feature Important for GoldenEye dataset.

Features name	Type	Feature Importance
Referrer	Text string	10
Query string	Text string	10
User-agent	Text string	9.276157329
Accept	Text string	3.520535328
Encoding	Text string	3.520535328
Source	IP string	0.748979997
Destination	IP string	0.748979997
Connection	Text string	0.748979997
Host	Text string	0.748979997

## Propose work

This research proposes a signature-based approach to detect the attack based on the extensive work in the problem analysis section and the feature selection process prepared in the methodology. This type of detection mechanism is suitable for the App DDoS detection, as the pattern is to randomly change false request headers. On the other hand, anomaly-based methods are more appropriate for unknown or very dynamic behaviors, and can suffer from false positives. While the forged request header might seem like a legitimate request header, it is not easily detectable. The attack can be effectively detected with the proper detection strategy. The detection algorithm is modular, that is, one algorithm will be sufficient for detecting the attack. The modular architecture enables the rules that are used for the detection to be updated, extended or refined without having to change the whole system, thus reducing maintenance complexity.

**Fig. 12:**
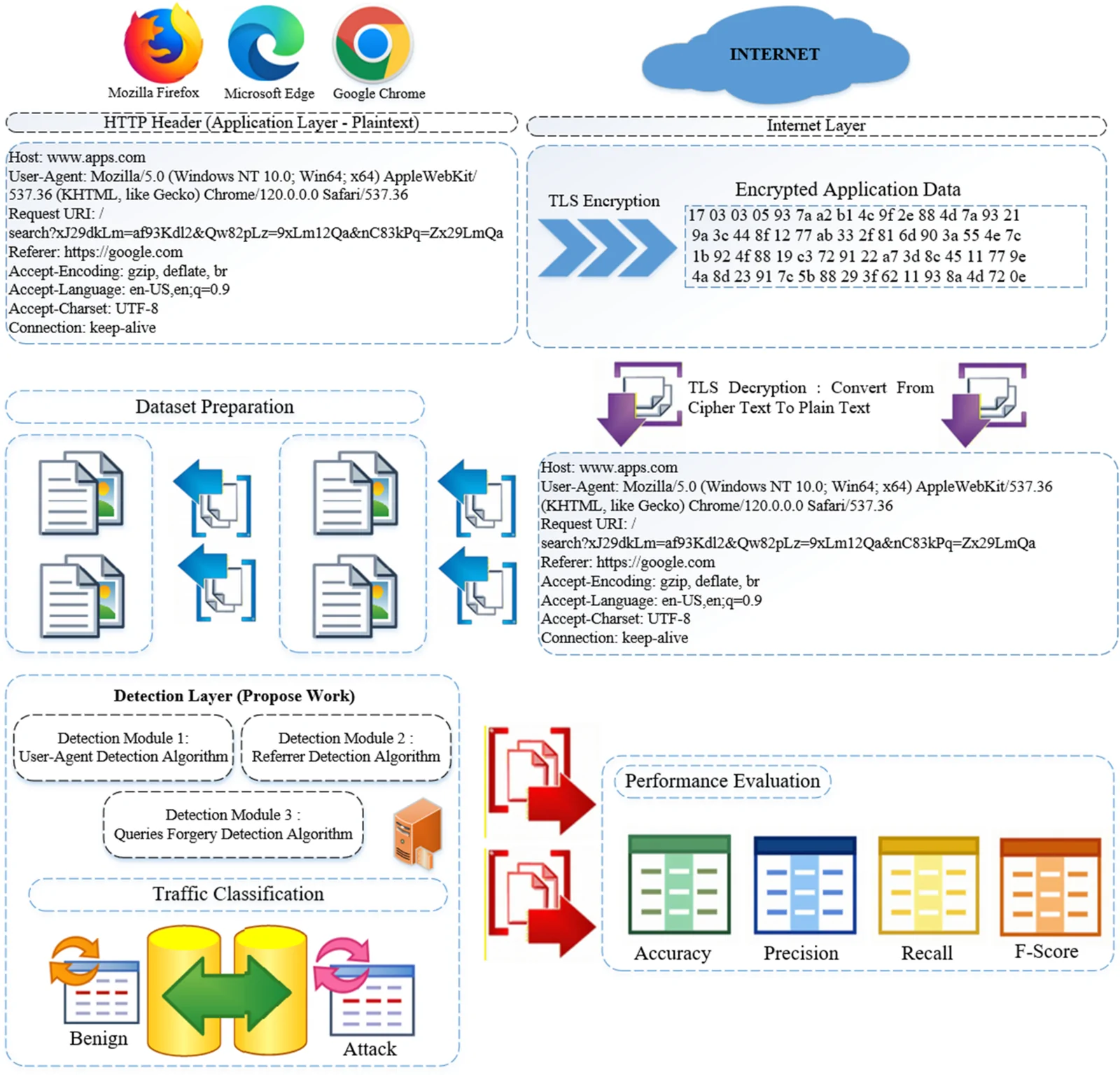
Detection framework.

This detection algorithm is designed to work with HTTP and HTTPS and will do header inspection after TLS decryption. In the HTTPS channel, traffic is encrypted and subsequently decrypted before the server processes the headers. Users primarily use a web browser to access online content. During this operation, the web browser includes a request header to inform the web server of the user’s request, and the request is generally transmitted over an encrypted channel. TLS performs request header decryption, and the availability of plaintext header information allows the server to determine how to respond. The detection framework is illustrated in Fig. 12, and the algorithm functionality is explained in Sect.  8.1 to 8.3.

### Forged user-agent detection algorithm

The algorithm classifies each user agent as legitimate or suspicious through a two-stage rule-based strategy. First, the algorithm parses the user agent string locally using the user agent’s parser to gather web browser information and the client type (mobile, desktop, tablet, or bot). Consequently, it applies internal validity rules to detect whether the raw string matches bot-crawler or scripting-client signatures. Next, the algorithm checks consistency by detecting mixed-platform token pairs (e.g., Windows and Mac OS X) and by identifying obsolete or improbable version tokens for browsers, operating systems, and rendering engines, including inconsistent WebKit, Safari, Chrome, and Trident/MSIE combinations. Besides that, the algorithm flags abnormal structures using length and spacing constraints and device-mismatch cues, such as an iPhone with Windows or a missing Android brand identifier, for mobile profiles. Algorithm 1 highlights the details of the forged user-agent detection.

**Figure:**
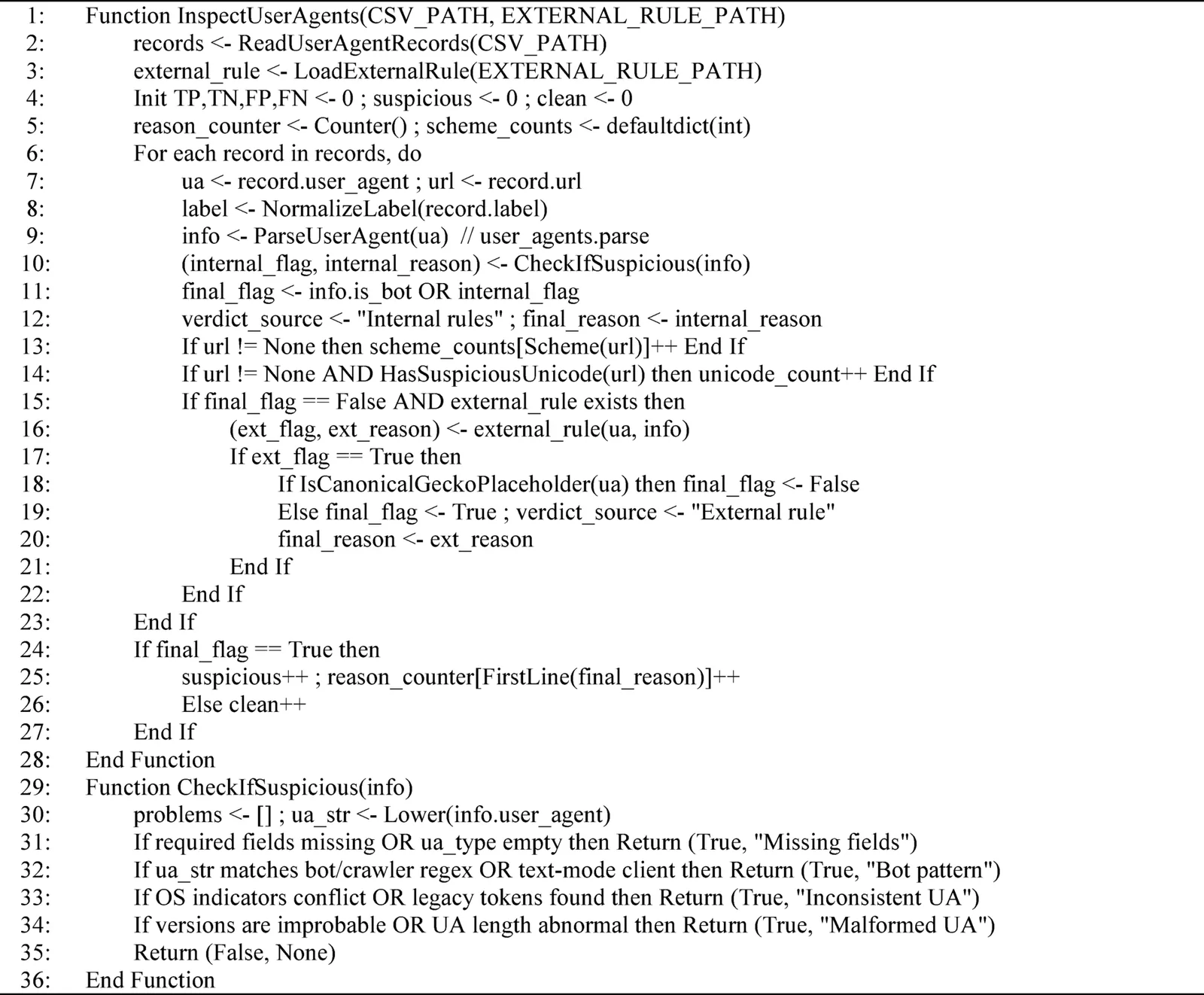
**Algorithm 1** User-agent detection algorithm.

### False referrer detection algorithm

The rerefer detection algorithm classifies referrers’ headers as suspicious or clean by combining deterministic validation rules with a lightweight heuristic risk score. The algorithm will parse the referrer URL and flag entries that fail parsing, lack a scheme or host, or use an unsupported scheme. Next, the algorithm inspects header consistency by comparing the referrer host to the origin host. Subsequently, the algorithm identifies bad content by detecting control characters, double-encoding indicators, and repeating or unusual Unicode byte patterns. Simultaneously, it calculates a heuristic score based on host and query signals, including embedded user information, raw IP addresses, excessive host length, substantial query parameters, excess percent-encoding, lengthy queries comparable to traversal paths, uncommon ports, and base-domain mismatches. Algorithm 2 illustrates the detection flow.

**Figure:**
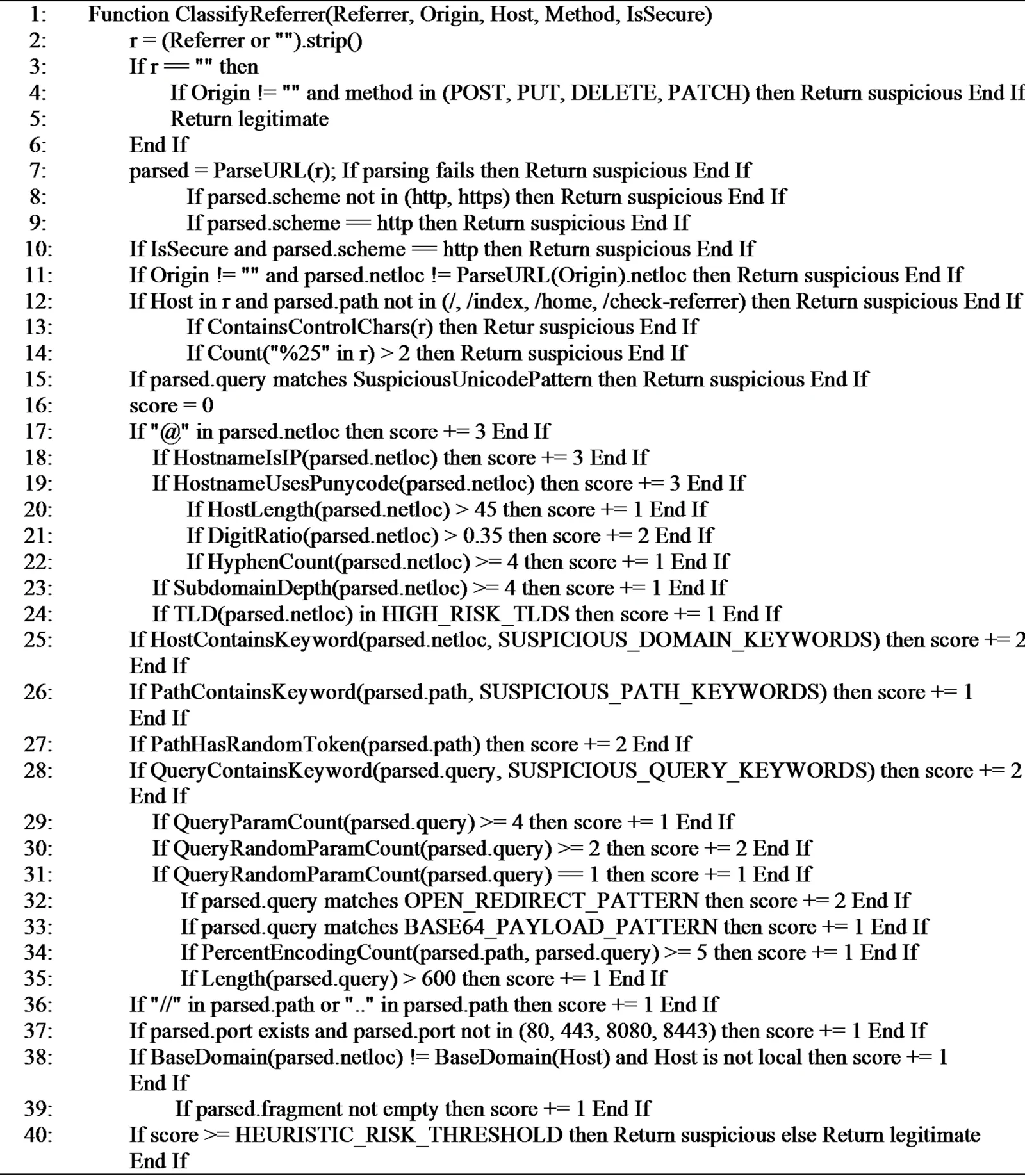
**Algorithm 2** Referrer detection algorithm.

### Queries forgery detection algorithm

The algorithm classifies query strings as legitimate or forged by removing whitespace, deleting a leading question mark, and decoding percent-encoded separators only when a percent sign precedes them. The subsequent phase identifies machine-like payloads by detecting non-ASCII byte escape sequences and a significant proportion of atypical characters beyond a standard URL character set. After the first screening, typical delimiters are used to tokenize the string into key-value pairs, and typical features are created, such as the number of parameters, the number of commas, and entropy. Legitimate traffic is determined when the query appears to be a URL and contains common human targets or common parameter keys with small, realistic values. The indicator of forgery queries is detected when parameters exhibit randomness through mixed-case numbers, when comma-separated key-value pairs exhibit high key entropy, or when numerous parameters exhibit high-entropy values. Algorithm 3 demonstrates the detection flow.

**Figure:**
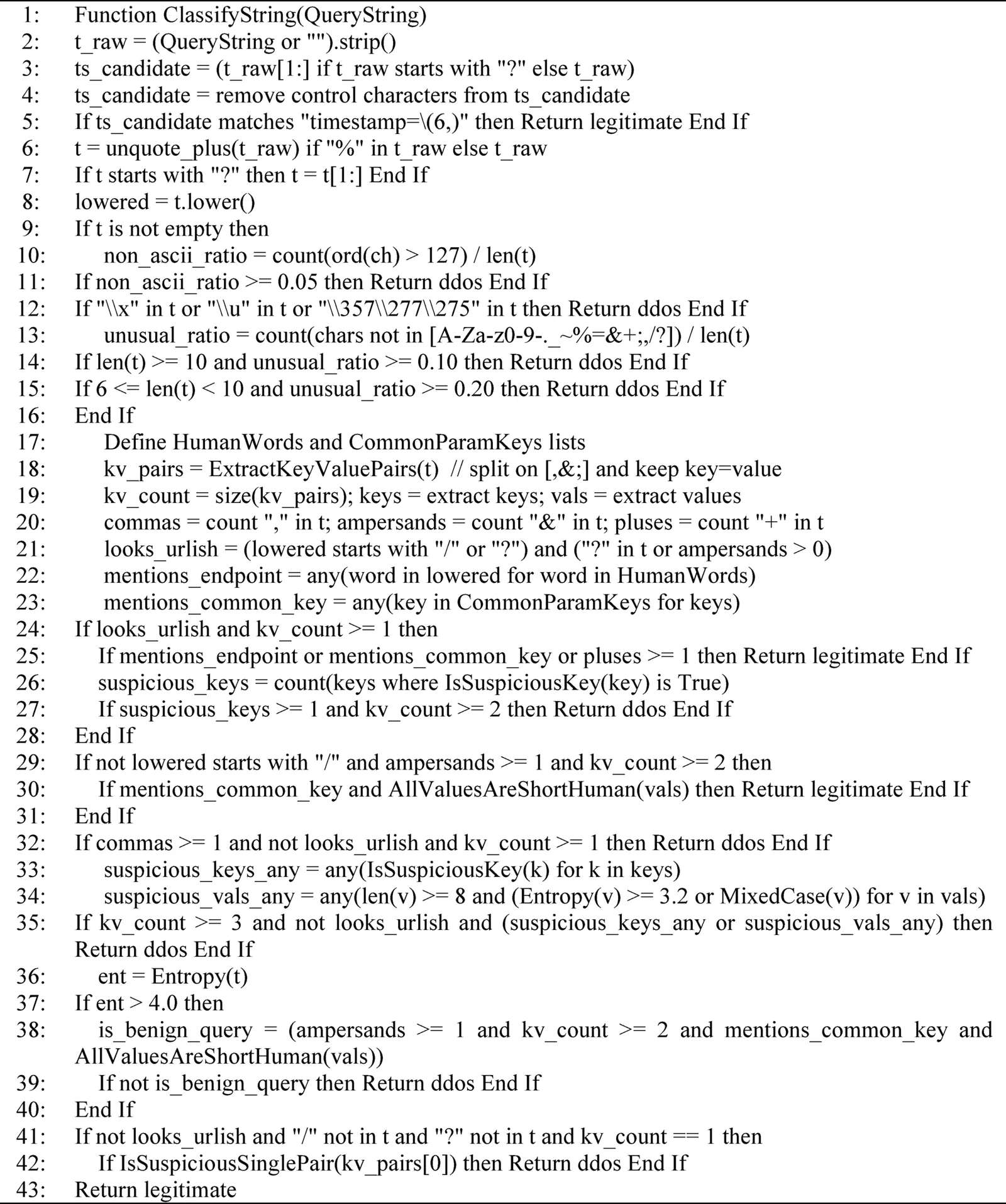
**Algorithm 3** Referrer detection algorithm.

## Results

The evaluation was conducted in three phases: the first phase evaluated the algorithm for forged user-agent detection, the second focused on forged referrer detection, and the last evaluated irrelevant query detection. Overall, the proposed detection algorithm efficiently recognized forged headers, though there was a slight drop during evaluation. However, through optimizing the detection rules, the performance was improved, and better results were obtained. The experiment is described as follows:

### Experiment 1: forged user agent detection

**Fig. 13:**
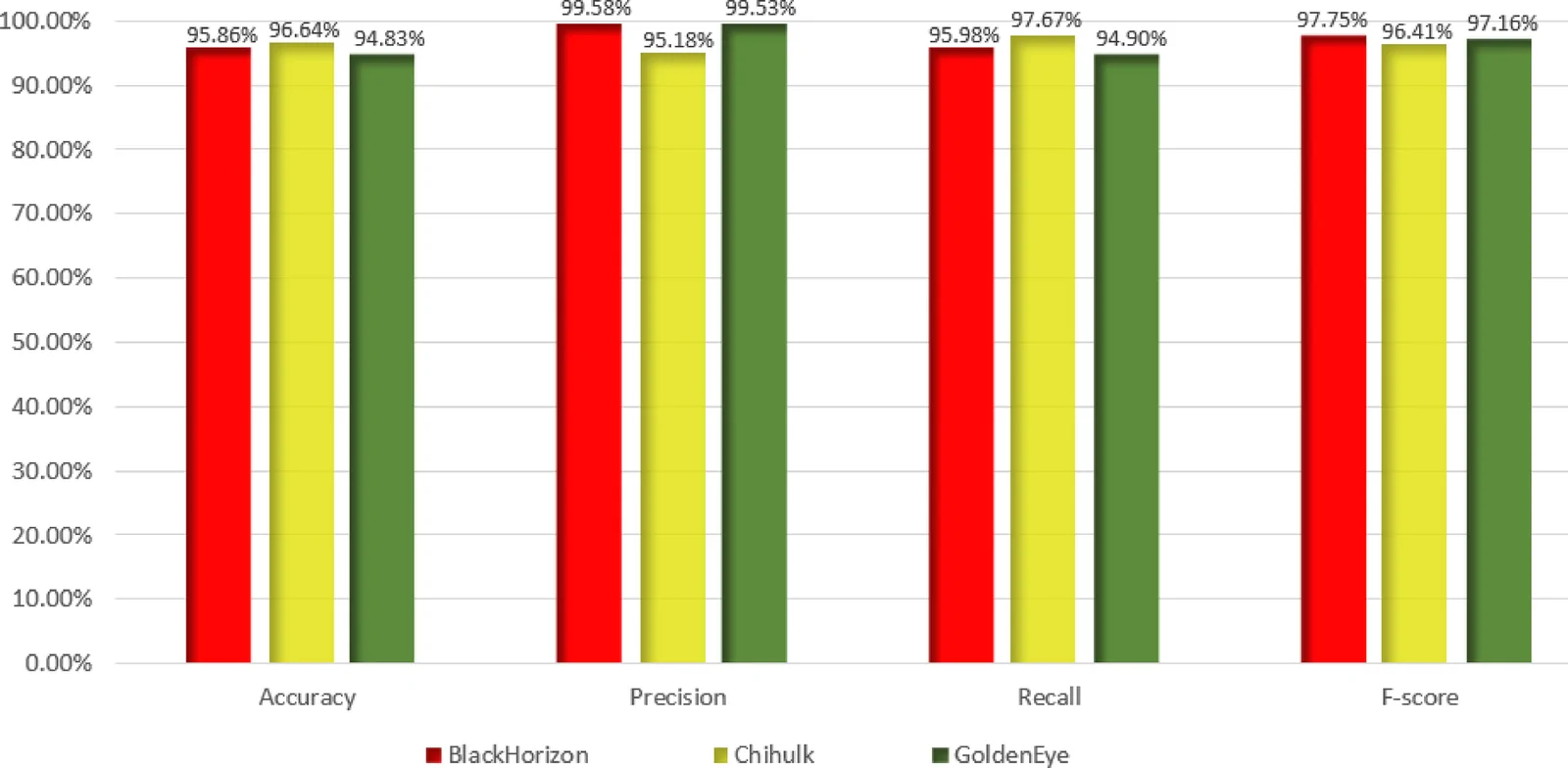
Detection performance for forged user agent.

As shown in Fig. 13, the detection capability is consistent in three App-DDoS datasets: BlackHorizon, ChiHulk, and GoldenEye. Among the proposed works, ChiHulk obtained the best accuracy of 96.64%, followed by BlackHorizon with 95.86% and GoldenEye with 94.83%. The accuracy achieved further underlines the viability of the suggested approach. BlackHorizon scored the top accuracy of 99.58%, and was closely trailed by GoldenEye with 99.53%. This means that the approach can reduce false-positive detections and accurately identify malicious requests. In the case of ChiHulk, a 97.67% recall was obtained, which means that it is very capable of detecting actual attacks. Based on overall precision and recall, BlackHorizon had the highest F-score of 97.75%, followed by GoldenEye with 97.16% and ChiHulk with 96.41%. The performance of the proposed detection model, as depicted in these results, is found to be consistent for various attack tools and traffic patterns.

### Experiment 2: forged referrer detection

To check whether the forgery and the authenticity classification are correct, the preliminary test was performed on all datasets in experiment 2, as the reference is bound to the URL, the structure of which is very complex. Further, this method allows them to detect a large number of URL patterns with the detection rule. The preliminary assessment results for the BlackHorizon dataset were 88.39% accuracy, 100% precision, 88.39% recall, and 93.84% F1-score. In Evaluation 1, the recall was 81.96%, and the F1-score was 90.09%. At the end of the evaluation, it achieved 96.93% accuracy, 99.11% precision, 97.55% recall, and 98.32% F1-score. These results show that the detection algorithm can effectively discover malicious URL patterns with a low false-alarm rate. The results of the detection performance are shown in Table 12.

**Table 12:** Detection performance – BlackHorizon dataset.

No.	Testing type	Accuracy	Precision	Recall	F1-score
1.	Preliminary test	88.39%	100.00%	88.39%	93.84%
2.	Evaluation 1	81.96%	100.00%	81.96%	90.09%
3.	Evaluation 2 (final evaluation)	96.93%	99.11%	97.55%	98.32%

A preliminary evaluation is done using the ChiHulk data set, which has an accuracy of 94.78%, precision of 100.00%, recall of 94.78%, and F1-score of 97.32% in the Preliminary Test of this experiment. Evaluation 1 produced a slight decrease in performance, with the accuracy dropping to 92.17% and, the recall dropping to 95.92%, and the F1-score dropping to 95.92%. However, overall accuracy was maintained at 100.00%. With the detection rules modified, the accuracy, precision, recall, and F1 score were 97.82%, 99.69%, 98.06% and 98.87%, respectively. The last evaluation shows that the false and correct URLs were recognized correctly. Results are shown in Table 13.

**Table 13:** Detection performance – ChiHulk dataset.

No.	Evaluation	Accuracy	Precision	Recall	F1-score
1.	Preliminary test	94.78%	100.00%	94.78%	97.32%
2.	Evaluation 1	92.17%	100.00%	92.17%	95.92%
3.	Evaluation 2 (final evaluation)	97.82%	99.69%	98.06%	98.87%

**Fig. 14:**
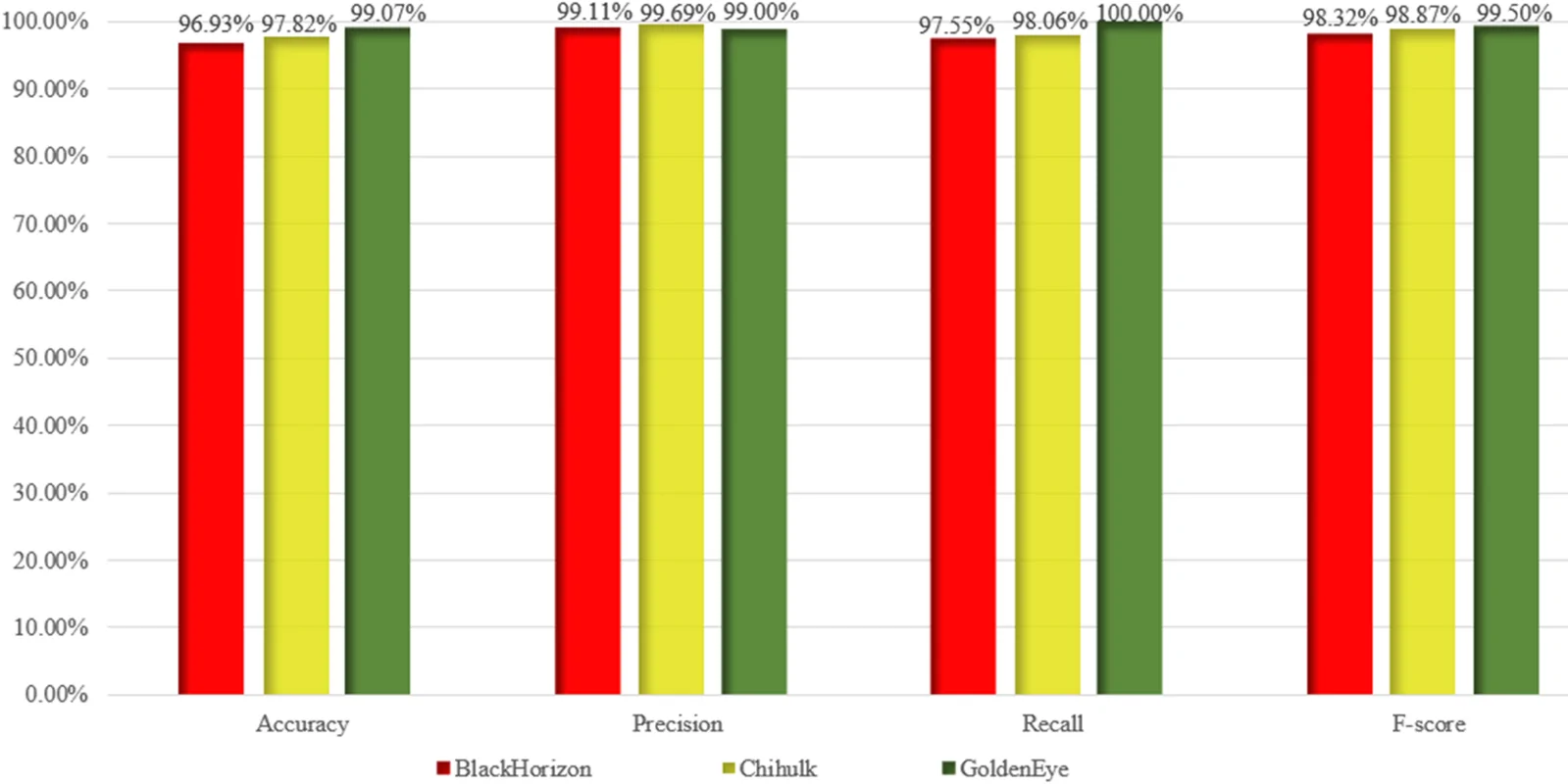
Detection performance for different datasets.

The preliminary assessment for the GoldenEye dataset was performed, and the accuracy of 99.53%, precision of 100.00%, recall of 99.53%, and F1 score of 99.77% were obtained. Nonetheless, a reduction in overall performance was noted for Evaluation 1, with accuracy and recall falling to 91.28% and the F1-score decreasing to 95.44%. Conversely, precision remained constant at 100.00%. The low accuracy and recall indicate the need to revise the detection criteria. Following the adjustment, the final assessment achieves optimal performance across all metrics. Table 14 presents the results, and Fig. 14 summarizes the final detection performance across the three datasets.

**Table 14:** Detection performance – GoldenEye Dataset.

No.	Evaluation	Accuracy	Precision	Recall	F1-score
1.	Preliminary test	99.53%	100.00%	99.53%	99.77%
2.	Evaluation 1	91.28%	100.00%	91.28%	95.44%
3.	Evaluation 2 (final evaluation)	99.07%	99.00%	100.00%	99.50%

### Experiment 3: irrelevant query detection

The initial evaluation for the BlackHorizon dataset shows consistent results across all performance metrics. However, the performance showed a slight drop in accuracy, to 97.86%, with precision remaining at 100.00%, recall at 97.86%, and F1-score at 98.92%. The performance increased in Evaluation 2 and was consistently reproduced in the final assessment (Evaluation 3) on the BlackHorizon dataset. A preliminary test was conducted on the Blackhorizon dataset only to assess detection performance and optimize detection rules prior to real data testing. In this case, the strategy was deemed suitable because the attack patterns generated from the datasets were almost the same. Table 15 shows the results for irrelevant queries detection using the BlackHorizon dataset.

**Table 15:** Query detection - BlackHorizon.

No.	Evaluation	Accuracy	Precision	Recall	F1-score
1.	Preliminary Test	100.00%	100.00%	100.00%	100.00%
2.	Evaluation 1	97.86%	100.00%	97.86%	98.92%
3.	Evaluation 2	99.91%	100.00%	99.90%	99.95%
4.	Evaluation 3 (final evaluation)	99.91%	100.00%	99.90%	99.95%

The evaluation was continued using the ChiHulk dataset in experiment 3. In Evaluation 1, the algorithm was not able to separate the benign from the false query, to get 0.00% accuracy, precision, recall, and F1-score. The accuracy of the Performance in Evaluation 2 was 2.21% with slight improvement. However, the precision, recall, and F1-score were still 0.00%. The algorithm’s performance improved significantly in the most recent evaluation (Evaluation 3) following the adjustment of the detection rule, attaining 100.00% accuracy, precision, recall, and F1-score. The detection performance is shown in Table 16.

**Table 16:** Query detection - ChiHulk.

No.	Evaluation	Accuracy	Precision	Recall	F1-score
1.	Evaluation 1	0.00%	0.00%	0.00%	0.00%
2.	Evaluation 2	2.21%	0.00%	0.00%	0.00%
3.	Evaluation 3 (final evaluation)	100.00%	100.00%	100.00%	100.00%

**Fig. 15:**
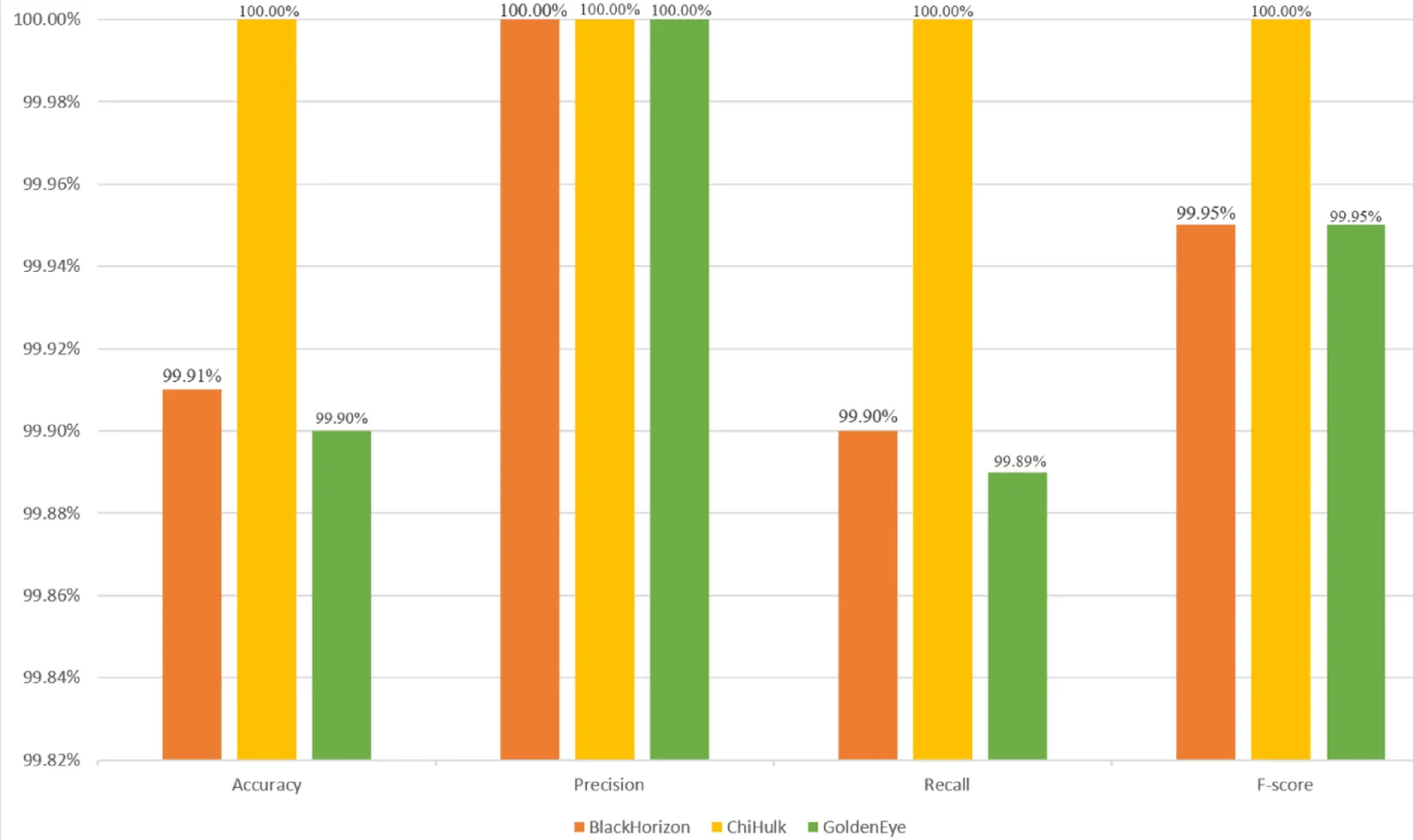
Performance graph for irrelevant query detection.

The algorithm has achieved an accuracy of 97.77%, precision of 100.00%, recall of 97.77%, and F1 score of 98.87% in Evaluation 1, when tested on the GoldenEye dataset in the last evaluation. The algorithm showed better performance during Evaluation 2 with 99.90% accuracy, 100.00% precision, 99.89% recall, and 99.95% F1 score. In the third evaluation (Evaluation 3), the same result was obtained. These results validate the detection algorithm’s stability and efficacy in attaining a high detection rate at all times. The results are given in Table 17, and a summary of the final detection performance in the three data sets is given in Fig. 15.

**Table 17:** Query detection - GoldenEye.

No	Evaluation	Accuracy	Precision	Recall	F1-score
1.	Evaluation 1	97.77%	100.00%	97.77%	98.87%
2.	Evaluation 2	99.90%	100.00%	99.89%	99.95%
3.	Evaluation 3 (final evaluation)	99.90%	100.00%	99.89%	99.95%

### Comparison

Results of comparison with previous research showed that the overall performance of detection was better than that of previous research. The second and third detection algorithms performed better, despite minor differences in performance metrics. The result comparison shows that the proposed detection algorithm can deal well with the request-header forgery of the App DDoS attack based on the real attack tools and automated attack tools reflecting the current attack trend. Many previous works use legacy benchmark sets, which may not represent the current real-world attack patterns due to the continuously changing attack patterns. Using older datasets for training and testing can lead to overestimation of the models since they lack diversity in behaviour, and the traffic is less complex. The comparison is shown in Table 18.

**Table 18:** Result comparison.

No.	Author	Accuracy	Precision	Recall	F1-Score
1.	Xie et al.,^75^	99.6%	–	99.3%	99.2%
2.	Mohammed Sharif and Beitollahi,^76^	99.9%	100%	99.8%	99.9%
3.	Kareem et al.,^77^	99.0%	99.5%	99.7%	99.6%
4.	Sharif,^78^	99.9%	99.9%	99.9%	99.9%
5.	Sharif,^79^	99.1%	92.2%	98.4%	95.0%
6.	Proposed work - forged user agent detection	95.86%	99.58%	95.98%	97.75%
	Proposed work - forged referrer detection	96.93%	99.11%	97.55%	98.32%
	Proposed work - irrelevant query detection	99.07%	99.00%	100.00%	99.50%

### Statistical analysis

**Fig. 16:**
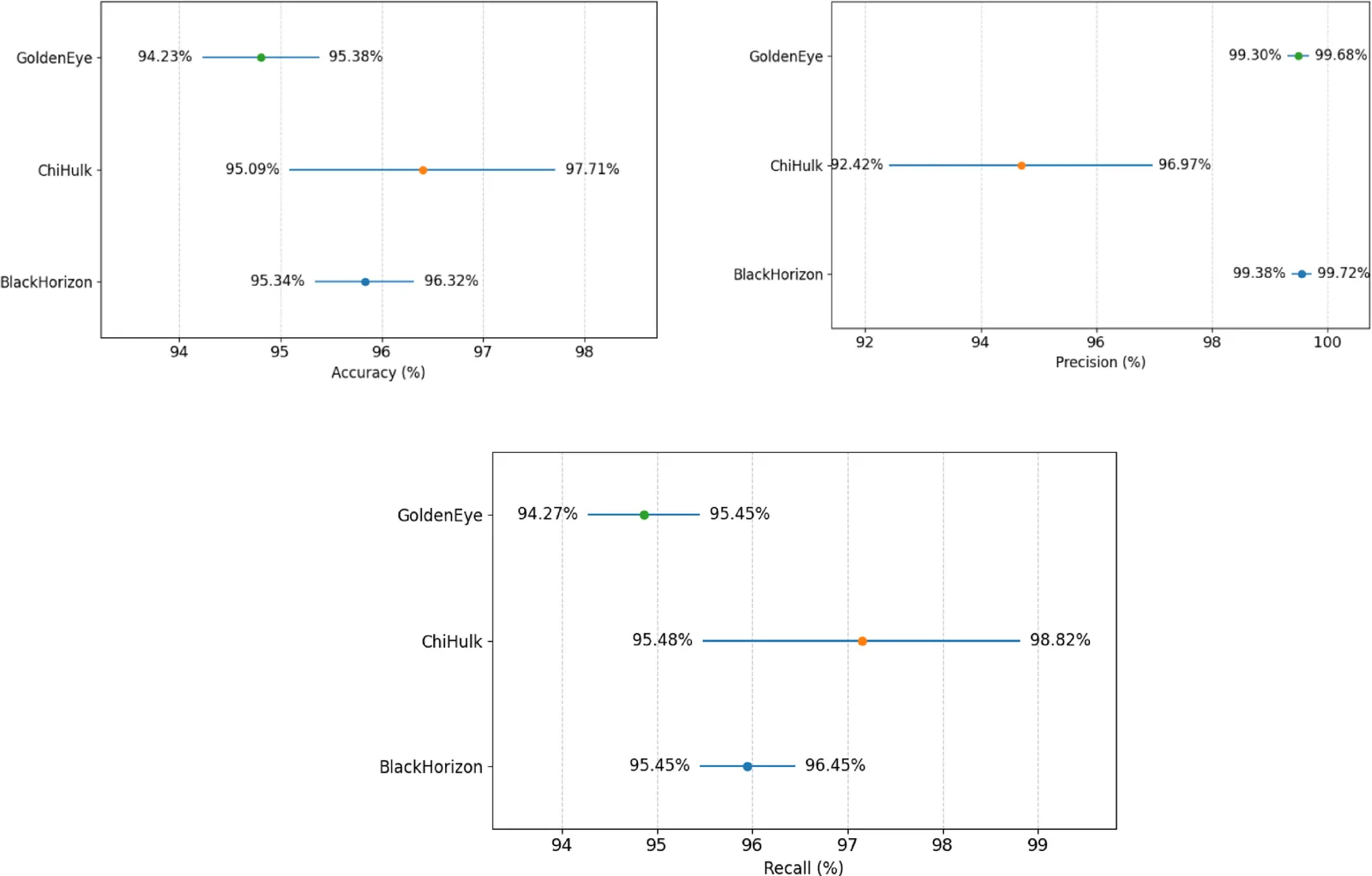
Confidence interval for forged user agent detection algorithm.

**Fig. 17:**
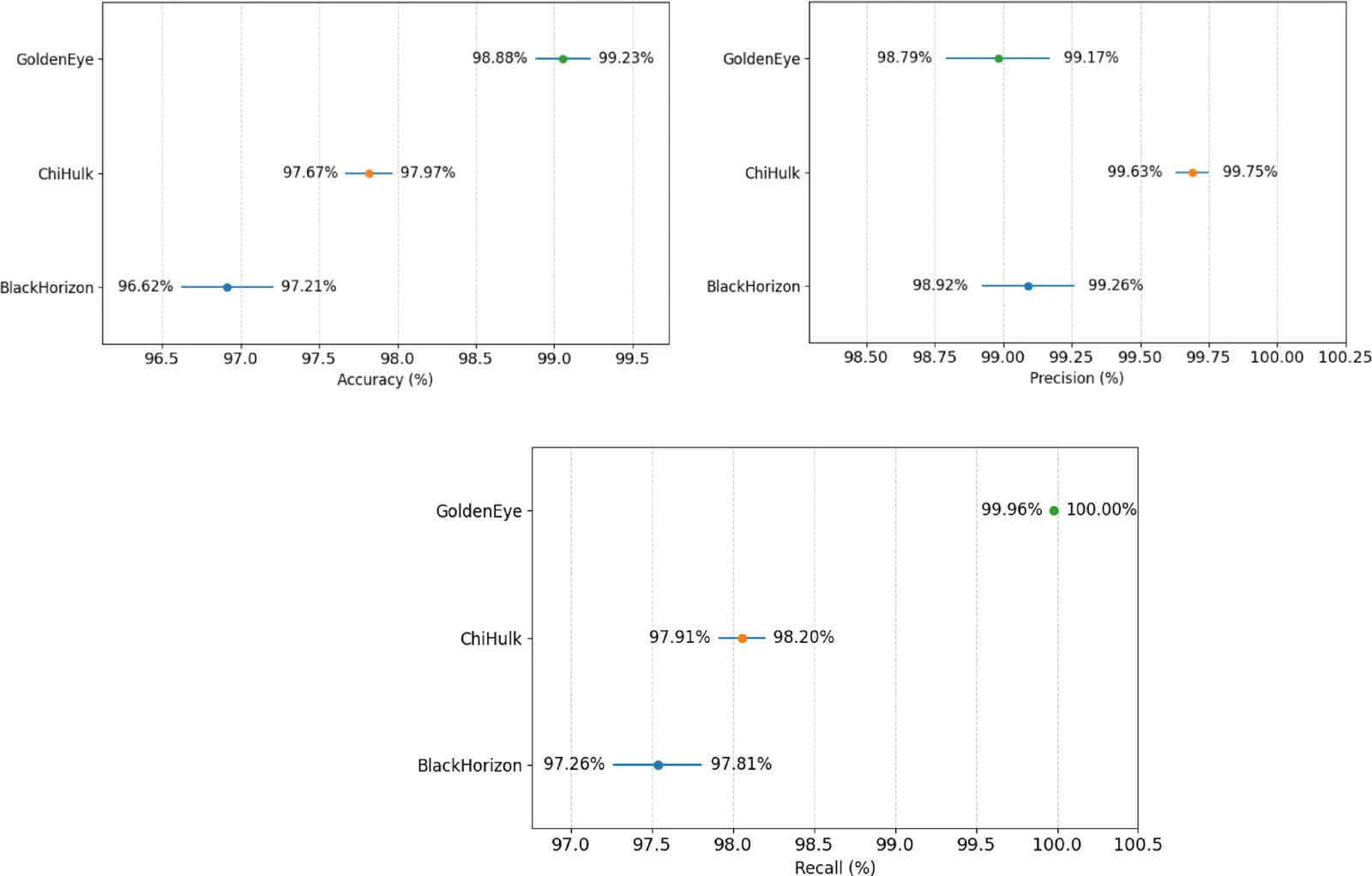
Confidence interval for forged referrer algorithm.

**Fig. 18:**
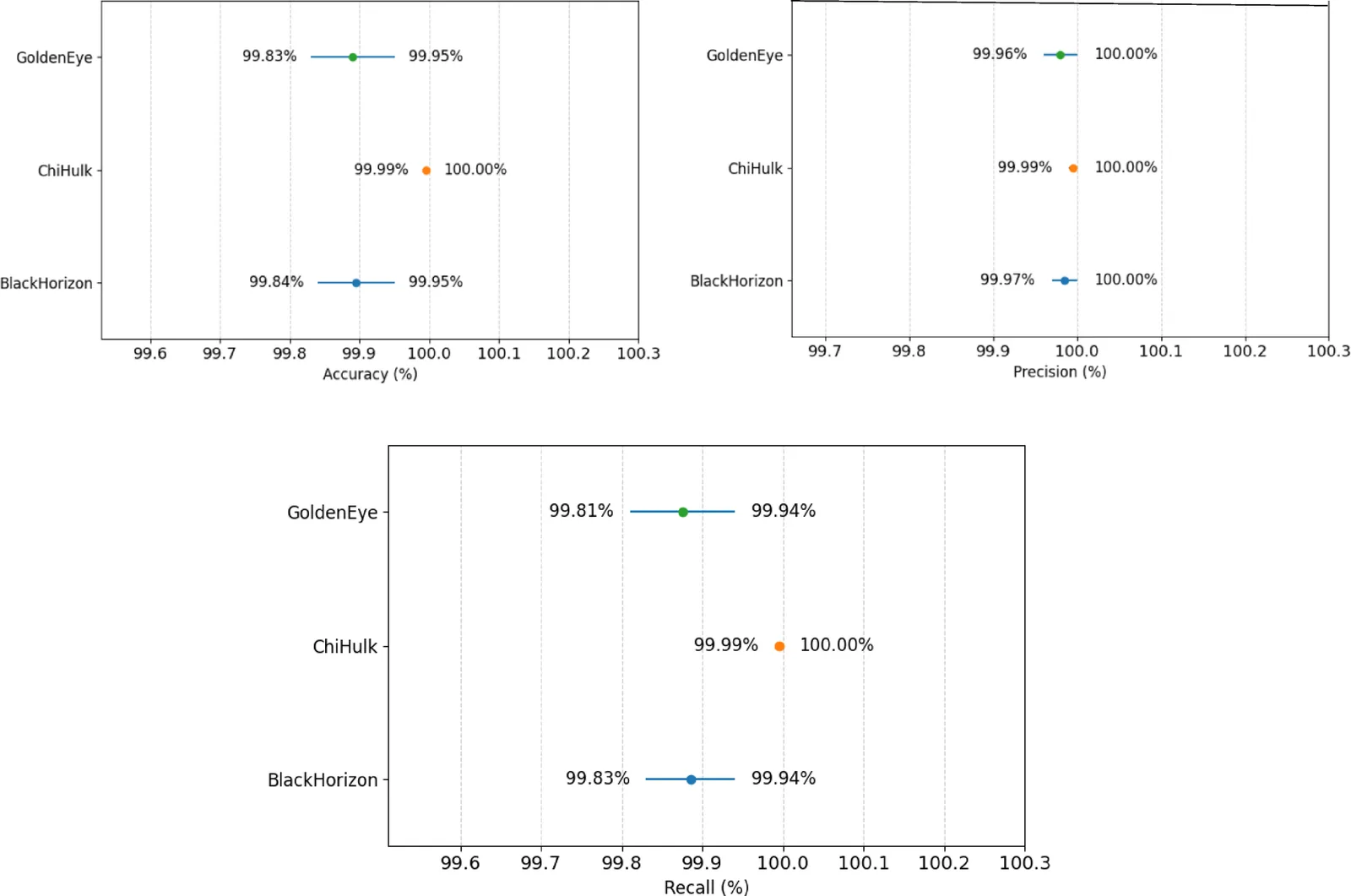
Confidence interval for irrelevant query detection.

To assess statistical reliability, 95% confidence intervals were calculated using the Wilson score method for accuracy, precision, and recall. This analysis is important because it provides an estimated performance range for each metric, rather than relying on a single metric. Based on the results, the forged user-agent detection algorithm achieves accuracy and recall intervals above 94% across all datasets. At the same time, precision remains very high for BlackHorizon and GoldenEye. The forged referrer detection algorithm shows stronger stability, with accuracy above 96%, precision above 98%, and recall reaching up to 100% for GoldenEye. The irrelevant query detection algorithm records the strongest performance, with accuracy, precision, and recall consistently approaching 100% across all datasets. Figures 16, 17 and 18 illustrate the results.

## Discussion

### Result

The forged user-agent detection algorithm achieves strong performance because the inspection-based detection strategy effectively distinguishes forged user agents from legitimate ones. App DDoS attacks use forged user agents that exhibit inconsistent user-agent structures, outdated versions, incomplete identifiers, version discrepancies, and web crawler signatures. Another important point to highlight is that the attack uses forged URLs and manipulated query strings, as further explained in Sect.  4. This situation highlights that developing signature-based detection rules requires a systematic approach to correctly recognize the attack. The multiple rounds of detection rule refinement are necessary to effectively distinguish legitimate from forged request headers (user-agent, referrer, query string). This process is conceptually comparable to the development cycle of anomaly-based detection, where ML models undergo training and testing phases to distinguish between attack and legitimate traffic patterns. The experimental results demonstrate that the signature-based detection strategy performs efficiently. Tables 19, 20 and 21 present the progressive evaluation outcomes during algorithm development and justify the algorithm’s high detection performance.

**Table 19:** Performance detection progression–forged user agent.

No.	Testing	Accuracy	Precision	Recall	F1-score
1.	Preliminary Test 1	91.50%	100.00%	83.00%	90.71%
2.	Preliminary Test 2	92.00%	92.86%	91.00%	91.92%
3.	Preliminary Test 3	98.00%	97.06%	99.00%	98.02%
4.	Improvement Test 2 from Test 1	0.50%	− 7.14%	8.00%	1.21%
5.	Improvement Test 3 from Test 1	6.50%	− 2.94%	16.00%	7.31%
6.	Improvement Test 3 from Test 2	6.00%	4.20%	8.00%	6.10%

**Table 20:** Performance detection progression–forged referrer.

No.	Dataset	Evaluation	Accuracy	Precision	Recall	F1-score
1.	BlackHorizon	Preliminary test	88.39%	100.00%	88.39%	93.84%
		Evaluation 1	81.96%	100.00%	81.96%	90.09%
		Evaluation 2 (final evaluation)	96.93%	99.11%	97.55%	98.32%
		Improvement from preliminary test	8.54%	− 0.89%	9.16%	4.48%
		Improvement from evaluation 1	14.97%	− 0.89%	15.59%	8.23%
2.	ChiHulk	Preliminary test	94.78%	100.00%	94.78%	97.32%
		Evaluation 1	92.17%	100.00%	92.17%	95.92%
		Evaluation 2 (final evaluation)	97.82%	99.69%	98.06%	98.87%
		Improvement from preliminary test	3.04%	− 0.31%	3.28%	1.55%
		Improvement from evaluation 1	5.65%	− 0.31%	5.89%	2.95%
3.	GoldenEye	Preliminary test	99.53%	100.00%	99.53%	99.77%
		Evaluation 1	91.28%	100.00%	91.28%	95.44%
		Evaluation 2 (final evaluation)	99.07%	99.00%	100.00%	99.50%
		Improvement from preliminary test	− 0.46%	− 1.00%	0.47%	− 0.27%
		Improvement from evaluation 1	7.79%	− 1.00%	8.72%	4.06%

**Table 21:** Performance detection progression–irrelevant string.

No.	Dataset	Evaluation	Accuracy	Precision	Recall	F1-score
1.	BlackHorizon	Preliminary test	100.00%	100.00%	100.00%	100.00%
		Evaluation 1	97.86%	100.00%	97.86%	98.92%
		Evaluation 2	99.91%	100.00%	99.90%	99.95%
		Evaluation 3 (final evaluation)	99.91%	100.00%	99.90%	99.95%
		Improvement from evaluation 1	2.05%	0.00%	2.04%	1.03%
		Improvement from evaluation 2	0.00%	0.00%	0.00%	0.00%
2.	ChiHulk	Evaluation 1	0.00%	0.00%	0.00%	0.00%
		Evaluation 2	2.21%	0.00%	0.00%	0.00%
		Evaluation 3 (final evaluation)	100.00%	100.00%	100.00%	100.00%
		Improvement from evaluation 1	100.00%	100.00%	100.00%	100.00%
		Improvement from evaluation 2	97.79%	100.00%	100.00%	100.00%
3.	GoldenEye	Evaluation 1	97.77%	100.00%	97.77%	98.87%
		Evaluation 2	99.90%	100.00%	99.89%	99.95%
		Evaluation 3 (final evaluation)	99.90%	100.00%	99.89%	99.95%
		Improvement from evaluation 1	2.13%	0.00%	2.12%	1.08%
		Improvement from evaluation 2	0.00%	0.00%	0.00%	0.00%

### Maintenance

In terms of maintenance requirements, both signature-based and anomaly-based detection mechanisms require continuous updates. Signature-based detection requires the detection rules to be updated whenever new attack vectors or evasion techniques emerge. Conversely, anomaly-based detection requires periodic retraining and revalidation of the ML models to accommodate distributional shifts, evolving authentic traffic behavior, and new emerging attack patterns^80,81^. Nonetheless, signature-based detection has several advantages, as the detection rules are fully customizable. Therefore, based on the observed attack pattern in the App DDoS attacks and the demonstrated results, this research concludes that signature-based detection is a suitable and robust approach for detecting header-level forgery produced by the attack.

### Detection capability

Most recent research focuses on anomaly detection as the primary method for dealing with cyberattacks, regardless of attack type. However, many studies overlook anomaly detection, which has several drawbacks, including vulnerability to data poisoning and model hijacking. More seriously, when hackers manipulate the model and the training data, the model becomes ineffective at detecting the attack. Signature‑based intrusion detection is efficient and highly interpretable, since decisions are based on explicit rules or known attack signatures. However, it cannot capture zero-day or emerging threats due to its ability to match only predefined patterns^82,83^. Recent AI‑based approaches, such as deep learning, anomaly detection, and federated learning, on the other hand, can learn complex patterns of behavior. It detects new attacks, thus enhancing its zero‑day detection capabilities^84–86^. However, this benefit is accompanied by some drawbacks, such as the “black box” nature of AI models, which can make them less interpretable than signature rules^87^. Another significant challenge is the requirement for extensive computational resources to train and test the data before generating a reliable model that can be deployed, given the demands of large-scale data training and the need to extract features for the model^88^. The deployment is also more complex, since AI‑based systems need constant retraining, the updating of datasets, and careful tuning to prevent false positives. However, signature‑based systems are easier to use. However, they require frequent manual updates of signatures^89^. Table 22 compares signature-based and AI-based detection.

**Table 22:** Comparison table signature and AI-based detection.

No.	Criteria	Signature-based detection	AI-based detection
1.	Interpretability	Highly interpretable because detection is based on explicit known signatures or byte patterns; decisions are deterministic and transparent^90^	Often low interpretability, especially deep‑learning‑based IDS, which operate as “black boxes,” requiring XAI for transparency^91^
2.	Detection of Zero-Day Patterns	Weak; cannot detect unseen or polymorphic threats because signatures must exist beforehand. Zero‑day recall is low^82,92–94^	Stronger capability; ML/DL anomaly detectors model normal or behavioral patterns to detect unseen attacks, including zero-day threats^95–97^
3.	Computational Cost	Low computational overhead due to simple pattern‑matching; efficient for real‑time deployment^90^	Often, there is a high computational overhead, especially for deep learning and ensemble models that require intensive training and inference. Lightweight AI exists, but it is still more demanding than signatures^98,99^
4.	Deployment Complexity	Low complexity: easy to deploy and maintain but requires continuous signature updates, which is labour-intensive^100,101^	Higher complexity due to training pipelines, feature engineering, model updates, explainability integration, and MLOps requirements^102^

AI detection can help security systems identify suspicious patterns, abnormal behavior, and potential cyberattacks, and can improve the detection of specific threats, such as SQL injection attacks, by learning malicious temporal and spatial patterns from network flow records^103^. However, it has limitations because the performance depends on data quality, model complexity, and real-time deployment capability. While AI significantly enhances threat detection, research indicates it still struggles with critical limitations such as feature extraction, generalizing to novel attacks, and managing imbalanced datasets, highlighting that high-quality training data remains essential for reliable security performance^104^. The AI detection models also require continuous monitoring and periodic retraining to remain effective against new attack patterns^105,84^. Given this atmosphere, signature-based detection is the ideal approach to address the forged headers utilized by App DDoS attacks.

### Methodology

Another important point is that the well-defined methodology employed in this research can be replicated in future studies to enhance the detection algorithms, thereby significantly reducing the time required to discover precise features. The code analysis conducted in this research demonstrates that static and dynamic analysis can identify the variables and features used by hackers to develop automated attack tools. Even though code analysis can yield good results, a well-defined methodology employed in this research can be replicated in future studies to improve. Unlike in Exploratory Data Analysis (EDA), when conducting statistical analysis, many tests must be run before finalizing features. Aside from that, even though these analyses can identify features that contribute to the attack, they do not fully capture the actual behavior of the attack or provide clear indicators of how the features within automated attack tools are utilized. This research uses three approaches to understand the characteristics of the attack: static, dynamic, and statistical, which justify the suitability of signature-based detection and describe the consistently high performance achieved by the proposed algorithm.

### Secure channel validation

The proposed work remains applicable in HTTPS environments because HTTP termination logically occurs before processing by the server. Modern systems routinely terminate TLS at reverse proxies or middleware to decrypt traffic for inspection^106,107^ and to enable the servers to process requests and generate responses. Nevertheless, the experiment could not validate the effectiveness of the proposed approach in encrypted channels, as this research focuses on detecting App DDoS attacks after decryption of request headers. Even though validation is not possible, the proposed approach can logically integrate with intrusion detection systems, web application firewalls, or reverse proxy environments, since these devices support modular security components and inline extension modules. Most modern security products adopt modular and add-on architectures. Recent research has shown that these concepts of modularity, extensible security layers, and plug-in mechanisms are now essential design principles in current cyber security systems^108,109^. Furthermore, web application firewalls function as reverse proxies and inspect decrypted Layer 7 traffic^110^, thereby making them natural integration points.

### The automated attack tools

The detection algorithm is based on the logical consistency of browser, operating system, and device information instead of just signatures. This way, the algorithm could distinguish a variety of modern web browsers and a forgery of them as long as headers are consistent, including those from mobile devices. Headers that are seemingly valid but include inconsistencies are often created using automated attack tools. Hence, the algorithm is developed to detect suspicious signs of mixed platforms, unrealistic combinations of versions, and unusual query behavior. This approach makes it possible for the detection algorithm to identify forged traffic even if the attackers try to mimic actual users, and it is not deceptive when intermediary devices are involved. Patterns are likely introduced by changes in browser technologies, mobile applications, and web standards that can affect detection accuracy, although the detection algorithms are effective. Hence, it is necessary to periodically update the detection rules to preserve the accuracy of the rules in a dynamic environment. However, despite anomaly detection powered by AI, the model needs to retrain to accommodate the new attack behaviors. Data patterns, browser behaviors, user interactions, and attack techniques are constantly evolving, necessitating regular updates to the cybersecurity detection models and rules^111,112^.

## Conclusion

In this study, the signature-based detection algorithm for App DDoS attack is advanced to identify App DDoS attack by inspecting the request header. The method was based on forged user-agent, referrer, and request-URI query patterns which were detected through static code analysis, dynamic code analysis, and statistical feature analysis. It is observed that these header fields are useful indicators for identifying automated App DDoS traffic in the datasets used in this study, which were controlled experimental datasets.

The results of the evaluation demonstrate that the proposed algorithm has strong detection performance on the BlackHorizon, ChiHulk, and GoldenEye datasets. After optimizing the rules, the forged referrer detection component and the irrelevant query detection component reached a high accuracy level, precision, recall, and F1-score. BlackHorizon and GoldenEye performed well with the forged user-agent component as well. This suggests that the detection performance might be dependent on the attack tool and header-generation strategy. The 95% Wilson confidence intervals of accuracy, precision, and recall showed that the approach proposed here had stable and consistent detection results on the evaluated data sets, with an interval of 95.28%, 97.97% for accuracy, 95.18%, 99.58% for precision, and 94.89%, 97.67% for recall. These are the findings that help support the evidence that the algorithm is reliable in the experimental conditions of this study. However, the findings of this research are still restricted to the controlled experimental setting and the datasets created by BlackHorizon, ChiHulk, and GoldenEye.

Although the proposed algorithm offers a good starting point for detecting forged-header patterns in automated App DDoS traffic, further testing should be conducted using recent production traffic, diverse types of benign traffic, and attack tools to fully evaluate its effectiveness when deployed in the real world. The study reveals that signature-based detection remains effective even when App DDoS attacks generate forged-header patterns.

## Future work

Signature-based detection is highly effective and accurate in detecting attacks that resemble the existing signatures or patterns^113,82,114,115^. Nevertheless, some research reports that the signature-based detection fails to detect new or zero-day attacks that cannot be matched with any signatures in the database^116–118^. This disadvantage can be overcome with a continuous rule update and assessments to make sure that it continues to identify new threats. Based on the results of the preliminary evaluation undertaken in this research and the final evaluation, it can be concluded that the proposed detection algorithm can solve the problem stated in Sect. 4. Hence, in the future, the automated rules to detect attacks and continuous evaluation mechanisms need to be explored to enhance the capability of the signature-based attack detection while also decreasing the amount of manual effort needed for rule creation and management by the administrator. The adoption of hybrid detection has the potential to mitigate the drawbacks of signature- and AI-based detection, and the exploration of temporal contrastive graph learning, as utilized by Wu et al.,^103^, could serve as an enhancement of this proposed work.

It is also important to note that most modern web services implement security in the data in transit and not at rest or in use. The TLS encryption does not eliminate the structure of HTTP requests. Instead, it encapsulates HTTP within an encrypted channel, such that the user-agent, referrer, host, and query string remain present at the application layer. App DDoS attacks continue to exploit these components by forging the header to evade detection. The proposed detection strategy remains valid in HTTPS environments, as it operates on the decrypted request header after TLS termination. Therefore, future research should focus on detecting the attack during data in transit, specifically during the encryption phase before it reaches a web server.

At this moment, the proposed detection algorithm is designed in a modular mode, and combining all the detection algorithms to detect App DDoS attacks is not feasible. The integration of all algorithms will be conducted in future work, with evaluation using larger, more diverse datasets, unseen attack tools, mutated attack variants, and deployment-based validation through edge gateways, web application firewalls, or reverse proxy environments.

## Supplementary Information

Below is the link to the electronic supplementary material.

Supplementary Material 1

Supplementary Material 2

Supplementary Material 3

## Data Availability

All data generated or analyzed during this study are included in this published article.

## References

[1] Bala, B. & Behal, S. AI techniques for IoT-based DDoS attack detection: Taxonomies, comprehensive review and research challenges. *Comput. Sci. Rev.* **52**, 100631. 10.1016/j.cosrev.2024.100631 (2024).

[2] Farias, E. P., de Neira, A. B., Borges, L. F. & Nogueira, M. Transformers model for DDoS attack detection: A survey. *Comput. Netw.* **270**, 111433. 10.1016/j.comnet.2025.111433 (2025).

[3] Li, Q. et al. A comprehensive survey on DDoS defense systems: New trends and challenges. *Comput. Netw.* **233**, 109895. 10.1016/j.comnet.2023.109895 (2023).

[4] Uddin, R., Kumar, S. A. P. & Chamola, V. Denial of service attacks in edge computing layers: Taxonomy, vulnerabilities, threats and solutions. *Ad Hoc Netw.* **152**, 103322. 10.1016/j.adhoc.2023.103322 (2024).

[5] Singh, C. & Jain, A. K. A comprehensive survey on DDoS attacks detection & mitigation in SDN-IoT network. *e-Prime - Adv. Electr. Eng. Electron. Energy*. **8**, 100543. 10.1016/j.prime.2024.100543 (2024).

[6] Ouhssini, M., Afdel, K., Akouhar, M., Agherrabi, E. & Abarda, A. Advancements in detecting, preventing, and mitigating DDoS attacks in cloud environments: A comprehensive systematic review of state-of-the-art approaches. *Egypt. Inf. J.* **27**, 100517. 10.1016/j.eij.2024.100517 (2024).

[7] Chahal, J. K., Bhandari, A. & Behal, S. DDoS attacks & defense mechanisms in SDN-enabled cloud: Taxonomy, review and research challenges. *Comput. Sci. Rev.* **53**, 100644. 10.1016/j.cosrev.2024.100644 (2024).

[8] netscout. NETSCOUT 5th Anniversary DDoS threat intelligence report. https://www.netscout.com/threatreport/2h2022/wp-content/uploads/2023/04/Threat-Report-2H2022.pdf (2022).

[9] Pacheco, O. Y. J. DDoS threat report for 2023 Q4. https://blog.cloudflare.com/ddos-threat-report-2023-q4/ (2024).

[10] Wang, S., Wu, H., Cheng, G., Hu, X. & Ren, J. SD-MDN-TM: A traceback and mitigation integrated mechanism against DDoS attacks with IP spoofing. *Comput. Netw.* **254**, 110793. 10.1016/j.comnet.2024.110793 (2024).

[11] Kaur, A., Rama Krishna, C. & Patil, N. V. A comprehensive review on Software-Defined Networking (SDN) and DDoS attacks: Ecosystem, taxonomy, traffic engineering, challenges and research directions. *Comput. Sci. Rev.* **55**, 100692. 10.1016/j.cosrev.2024.100692 (2025).

[12] Zineddine, A., Belfaik, Y., Sadqi, Y. & Safi, S. A novel hybrid cybersecurity assessment methodology for HTTPS deployment. *High-Confidence Comput.* 100344. 10.1016/j.hcc.2025.100344 (2025).

[13] Zineddine, A. et al. A systematic review of cybersecurity assessment methods for HTTPS.*Comput. Electr. Eng.*, **115**, 109137. 10.1016/j.compeleceng.2024.109137 (2024).

[14] Mohammadi, R., Lal, C., Conti, M. & Sharma, L. Software defined network-based HTTP flooding attack defender. *Comput. Electr. Eng.* **101**, 108019. 10.1016/j.compeleceng.2022.108019 (2022).

[15] Praseed, A. & Thilagam, P. S. HTTP request pattern based signatures for early application layer DDoS detection: A firewall agnostic approach. *J. Inform. Secur. Appl.*10.1016/j.jisa.2021.103090 (2022). 65.

[16] Gadient, P., Nierstrasz, O. & Ghafari, M. Security header fields in HTTP clients. In *2021 IEEE 21st International Conference on Software Quality, Reliability and Security (QRS)*, (2021).

[17] Del-Pozo-Puñal, E., Garcia-Carballeira, F., Camarmas-Alonso, D. & Calderon-Mateos, A. Hierarchical and distributed data storage for computing continuum. *Future Generation Comput. Syst.*, **174**, 107931. 10.1016/j.future.2025.107931 (2026).

[18] Zhou, K., Wu, T. & Zhang, J. EGOP: A Server-Side Enhanced Architecture to Eliminate End-to-End Latency Caused by GOP Length in Live Streaming. *Computers Mater. Continua*. **86** (1), 1–27. 10.32604/cmc.2025.068160 (2025).

[19] Huang, H., Ye, P., Hu, M. & Wu, J. A multi-point collaborative DDoS defense mechanism for IIoT environment. *Digit. Commun. Networks*. **9** (2), 590–601. 10.1016/j.dcan.2022.04.008 (2023).

[20] Chaudhary, P., Singh, A. K. & Gupta, B. B. Dynamic multiphase DDoS attack identification and mitigation framework to secure SDN-based fog-empowered consumer IoT Networks. *Comput. Electr. Eng.* **123**, 110226. 10.1016/j.compeleceng.2025.110226 (2025).

[21] Hawelitschek, M. et al. Digital transformation of a real-life R&D process using a structured approach based on the Internet of Things. *SLAS Technol.* **35**, 100359. 10.1016/j.slast.2025.100359 (2025). 41175974 10.1016/j.slast.2025.100359

[22] Maniriho, P., Mahmood, A. N. & Chowdhury, M. J. M. A systematic literature review on Windows malware detection: Techniques, research issues, and future directions. *J. Syst. Softw.* **209**, 111921. 10.1016/j.jss.2023.111921 (2024).

[23] Einy, S., Oz, C. & Navaei, Y. D. The anomaly-and signature-based IDS for network security using hybrid inference systems. *Math. Probl. Eng.* **2021**, 1–10 (2021).

[24] Jmila, H. & Khedher, M. I. Adversarial machine learning for network intrusion detection: A comparative study. *Comput. Netw.* **214**, 109073 (2022).

[25] Schmitt, M. Securing the Digital World: Protecting smart infrastructures and digital industries with Artificial Intelligence (AI)-enabled malware and intrusion detection. *J. Industrial Inform. Integr.* **36**, 100520 (2023).

[26] Myint Oo, M., Kamolphiwong, S., Kamolphiwong, T. & Vasupongayya, S. Advanced support vector machine- (ASVM-) based detection for distributed denial of service (DDoS) Attack on Software Defined Networking (SDN). *J. Comput. Networks Commun.* 1–12. 10.1155/2019/8012568 (2019).

[27] Gonçalves, D. S. M., Couto, R. S. & Rubinstein, M. G. A protection system against HTTP flood attacks using software defined networking. *J. Netw. Syst. Manage.* **31** (1). 10.1007/s10922-022-09704-1 (2022).

[28] Park, S., Kim, Y., Choi, H., Kyung, Y. & Park, J. HTTP DDoS flooding attack mitigation in software-defined networking. *IEICE Trans. Inform. Syst. E104 D*. **9**, 1496–1499. 10.1587/transinf.2021EDL8022 (2021).

[29] Patidar, A. & Somani, G. Serving while attacked: DDoS attack effect minimization using page separation and container allocation strategy. *J. Inform. Secur. Appl.* 59. 10.1016/j.jisa.2021.102818 (2021).

[30] Rao Varre, D. N. M. & Bayana, J. A Secured Botnet Prevention Mechanism for HTTP Flooding Based DDoS Attack 2022 3rd International Conference for Emerging Technology (INCET), (2022).

[31] Zhao, X. et al. Defending application layer DDoS attacks via multidimensional parallelotope. *Secur. Commun. Netw.* 6679304. 10.1155/2020/6679304 (2020).

[32] Sarker, I. H., Furhad, M. H. & Nowrozy, R. Ai-driven cybersecurity: an overview, security intelligence modeling and research directions. *SN Comput. Sci.* **2** (3), 173 (2021).

[33] Al-gethami, W. & Aljuhani, A. Detection of http attacks using machine learning. In *2022 2nd International Conference on Computing and Information Technology (ICCIT)*, (2022).

[34] Alghazzawi, D., Bamasag, O., Ullah, H. & Asghar, M. Z. Efficient detection of DDoS attacks using a hybrid deep learning model with improved feature selection. *Appl. Sci.* **11** (24), 11634 (2021).

[35] Mohammadi, R., Lal, C. & Conti, M. HTTPScout: A Machine Learning based Countermeasure for HTTP Flood Attacks in SDN. *Int. J. Inf. Secur.*10.1007/s10207-022-00641-3 (2022).

[36] Feng, Y., Li, J. & Nguyen, T. Application-Layer DDoS Defense with Reinforcement Learning. In *2020 IEEE/ACM 28th International Symposium on Quality of Service (IWQoS)*, (2020).

[37] Praseed, A. & Thilagam, P. S. Modelling behavioural dynamics for asymmetric application layer DDoS detection. *IEEE Trans. Inf. Forensics Secur.* **16**, 617–626. 10.1109/tifs.2020.3017928 (2021).

[38] Abubakar, R. et al. An Effective Mechanism to Mitigate Real-Time DDoS Attack. *IEEE Access.* **8**, 126215–126227. 10.1109/access.2020.2995820 (2020).

[39] Yu, X. et al. WEB DDoS Attack Detection Method Based on Semisupervised Learning. *Secur. Communication Networks*. **2021**, 1–10. 10.1155/2021/9534016 (2021).

[40] Beitollahi, H., Sharif, D. M. & Fazeli, M. Application Layer DDoS Attack Detection Using Cuckoo Search Algorithm-Trained Radial Basis Function. *IEEE Access.* **10**, 63844–63854. 10.1109/access.2022.3182818 (2022).

[41] Ahmetoglu, H. & Das, R. A comprehensive review on detection of cyber-attacks: Data sets, methods, challenges, and future research directions. *Internet Things*. **20**, 100615. 10.1016/j.iot.2022.100615 (2022).

[42] Díaz-Verdejo, J. E., Alonso, E., Estepa Alonso, R., Madinabeitia, G. & A., & A critical review of the techniques used for anomaly detection of HTTP-based attacks: taxonomy, limitations and open challenges. *Comput. Secur.* 124. 10.1016/j.cose.2022.102997 (2023).

[43] Shafi, M., Lashkari, A. H., Rodriguez, V. & Nevo, R. Toward generating a new cloud-based distributed denial of service (DDoS) dataset and cloud intrusion traffic characterization [Article]. *Inform. (Switzerland)*, **15**(4), Article 195. 10.3390/info15040195 (2024).

[44] Ghafar, A., Jaafar, Abdullah, S. M. & Adli, S. A review of technique to self-generate DDoS dataset. *Int. J. Adv. Trends Comput. Sci. Eng.* **8** (4). 10.30534/ijatcse/2019/88842019 (2019).

[45] Vashishtha, L. K. & Chatterjee, K. Strengthening cybersecurity: TestCloudIDS dataset and SparkShield algorithm for robust threat detection [Article]. *Comput. Secur.*, **151**, Article 104308. 10.1016/j.cose.2024.104308 (2025).

[46] Alhijawi, B., Almajali, S., Elgala, H., Salameh, H. B. & Ayyash, M. A survey on DoS/DDoS mitigation techniques in SDNs: Classification, comparison, solutions, testing tools and datasets. *Comput. Electr. Eng.* **99**, 107706 (2022).

[47] Kenyon, A., Deka, L. & Elizondo, D. Are public intrusion datasets fit for purpose characterising the state of the art in intrusion event datasets. *Comput. Secur.* **99**10.1016/j.cose.2020.102022 (2020).

[48] Zhang, C. et al. Comparative research on network intrusion detection methods based on machine learning. *Comput. Secur.* **121**. 10.1016/j.cose.2022.102861 (2022).

[49] Bahashwan, A. A. et al. HLD-DDoSDN: High and low-rates dataset-based DDoS attacks against SDN. *PLOS One* **19**(2), e0297548. 10.1371/journal.pone.0297548 (2024). 10.1371/journal.pone.0297548 PMC10852331 38330004

[50] Karmous, N., Jlassi, W., Aoueileyine, M. O. E., Filali, I. & Bouallegue, R. A New Dataset for Network Flooding Attacks in SDN-Based IoT Environments. *CMES - Comput. Model. Eng. Sci.* **145** (3), 4363–4393. 10.32604/cmes.2025.074178 (2025).

[51] Catillo, M., Pecchia, A. & Villano, U. No more DoS? An empirical study on defense techniques for web server Denial of Service mitigation. *J. Netw. Comput. Appl.* **202**, 103363. 10.1016/j.jnca.2022.103363 (2022).

[52] Saiyed, M. F. & Al-Anbagi, I. Flow and unified information-based DDoS attack detection system for multi-topology IoT networks. *Internet Things*. **24**, 100976. 10.1016/j.iot.2023.100976 (2023).

[53] Sinha, M. A comprehensive survey of DDoS attack defense systems for different SDN architectures. *Comput. Netw.* **272**, 111711. 10.1016/j.comnet.2025.111711 (2025).

[54] Alhayani, S. & Murphy, D. R. A Machine Learning-Based Distributed Denial of Service Detection Approach for Early Warning in Internet Exchange Points. *Computers Mater. Continua*. **76** (2), 2235–2259. 10.32604/cmc.2023.038003 (2023).

[55] Kaur, S., Kumar, K. & Aggarwal, N. Enhancing DDoS defense in SDN using hierarchical machine learning models. *J. Netw. Comput. Appl.* **239**, 104168. 10.1016/j.jnca.2025.104168 (2025).

[56] Muduli, D., Gupta, R. K., Majhi, S. K., Ojha, B. & Majhi, B. An optimized learning approach for enhancing the security of digital twin-enabled industrial systems from distributed denial-of-service attacks. *J. Industrial Inform. Integr.* **48**, 100960. 10.1016/j.jii.2025.100960 (2025).

[57] Pasha, M. J., Rao, K. P., MallaReddy, A. & Bande, V. LRDADF: An AI enabled framework for detecting low-rate DDoS attacks in cloud computing environments. *Measurement: Sens.* **28**, 100828. 10.1016/j.measen.2023.100828 (2023).

[58] Sheikh, M. N. A. et al. SDN-enabled IoT based transport layer DDoS Attacks detection using RNNs. *Comput. Mater. Continua* **85**(2), 4043–4066. 10.32604/cmc.2025.065850 (2025).

[59] Turkoglu, M., Polat, H., Kocak, C. & Polat, O. Recognition of DDoS attacks on SD-VANET based on combination of hyperparameter optimization and feature selection. *Expert Syst. Appl.* **203**, 117500. 10.1016/j.eswa.2022.117500 (2022).

[60] Yue, M., Yan, H., Han, R. & Wu, Z. A DDoS attack detection method based on IQR and DFFCNN in SDN. *J. Netw. Comput. Appl.* **240**, 104203. 10.1016/j.jnca.2025.104203 (2025).

[61] Berlin, J. https://github.com/N0taN3rd/userAgentLists/tree/master.

[62] George, E. & S, K. Hybrid SSA-GWO optimized ensemble learning for malicious node detection in wireless sensor networks. *Phys. Communication*. **76**, 103064. 10.1016/j.phycom.2026.103064 (2026).

[63] Jiang, H. et al. FUSE-IS: multi-modal data fusion for carbon-aware security in industrial energy systems. *Inform. Fusion*. **127**, 103759. 10.1016/j.inffus.2025.103759 (2026).

[64] Jelodar, H. et al. Large language model (LLM) for software security: Code analysis, malware analysis, reverse engineering. *J. Inform. Secur. Appl.* **98**, 104390. 10.1016/j.jisa.2026.104390 (2026).

[65] Pinto, D., Amorim, I., Maia, E. & Praça, I. A review on intrusion detection datasets: tools, processes, and features. *Comput. Netw.* **262**, 111177. 10.1016/j.comnet.2025.111177 (2025).

[66] Aceto, G. et al. Synthetic and privacy-preserving traffic trace generation using generative AI models for training Network Intrusion Detection Systems. *J. Netw. Comput. Appl.* **229**, 103926. 10.1016/j.jnca.2024.103926 (2024).

[67] Dong, J., Chen, H., Shen, S., Xu, H. & Liu, Z. Interpretable intrusion detection for IoT security: A SHAP-enhanced NGBoost model. *Comput. Netw.* **275**, 111937. 10.1016/j.comnet.2025.111937 (2026).

[68] Harish, M. S., Lokesh, S., Sakthivel, P. & Akshaya, B. Hybrid deep learning model for network intrusion detection using optimal feature fusion. *Ain Shams Eng. J.* **17** (1), 103904. 10.1016/j.asej.2025.103904 (2026).

[69] Kumar, V., Kumar, K., Singh, M. & Kumar, N. NIDS-DA: Detecting functionally preserved adversarial examples for network intrusion detection system using deep autoencoders. *Expert Syst. Appl.* **270**, 126513. 10.1016/j.eswa.2025.126513 (2025).

[70] Wang, P. et al. Data-driven software defined network attack detection: State-of-the-art and perspectives. *Inf. Sci.* **513**, 65–83. 10.1016/j.ins.2019.08.047 (2020).

[71] Alqurashi, F. & Ahmad, I. A data-driven multi-perspective approach to cybersecurity knowledge discovery through topic modelling. *Alexandria Eng. J.* **107**, 374–389. 10.1016/j.aej.2024.07.044 (2024).

[72] Che Yahaya, C. A., Firdaus, A., Zabidi, A., Ab Razak, M. F. & Narudin, F. A. Wordcloud: Detecting mobile malware with deep learning and word of cloud. *Egypt. Inf. J.* **32**, 100834. 10.1016/j.eij.2025.100834 (2025).

[73] Sharma, T. et al. A survey on machine learning techniques applied to source code. *J. Syst. Softw.* **209**, 111934. 10.1016/j.jss.2023.111934 (2024).

[74] Vidal, F. R., Ivaki, N. & Laranjeiro, N. Vulnerability detection techniques for smart contracts: A systematic literature review. *J. Syst. Softw.* **217**, 112160. 10.1016/j.jss.2024.112160 (2024).

[75] Xie, B., Wang, Y., Wen, G. & Xu, X. Application-Layer DDoS Attack Detection Using Explicit Duration Recurrent Network-Based Application-Layer Protocol Communication Models. *Int. J. Intell. Syst.* **2023** (1), 2632678. 10.1155/2023/2632678 (2023).

[76] Mohammed Sharif, D. & Beitollahi, H. Detection of application-layer DDoS attacks using machine learning and genetic algorithms. *Computers Secur.* **135**, 103511. 10.1016/j.cose.2023.103511 (2023).

[77] Kareem, M. K., Aborisade, O. D., Onashoga, S. A., Sutikno, T. & Olayiwola, O. M. Efficient model for detecting application layer distributed denial of service attacks. *Bull. Electr. Eng. Inf.* **12** (1), 441–450 (2023).

[78] Sharif, D. M. Application-layer DDoS detection via efficient machine learning and feature selection. In *2023 International Conference on Engineering Applied and Nano Sciences (ICEANS) *(2023).

[79] Sharif, D. M. High-accurate application-layer DDoS attack detection using machine learning. In *2023 International Conference on Engineering Applied and Nano Sciences (ICEANS)*, (2023).

[80] Abdoune, F., Nouiri, M. & Cardin, O. Digital twin-based anomaly detection under concept drift: A comparison between iterative batch learning and incremental learning approaches. *Expert Syst. Appl.* **299**, 130062. 10.1016/j.eswa.2025.130062 (2026).

[81] Shi, Y. et al. A review of deep learning based anomaly detection. *Neurocomputing* **668**, 132383. 10.1016/j.neucom.2025.132383 (2026).

[82] Aydin, B., Aydin, H. & Gormus, S. Intrusion detection systems in IoT: A detailed review of threat categories, detection strategies, and future technologies. *J. Inform. Secur. Appl.* **95**, 104291. 10.1016/j.jisa.2025.104291 (2025).

[83] Zhang, J. et al. Enhancing network intrusion detection with adaptive spatial convolution and salient contrastive learning. *Comput. Netw.* **281**, 112226. 10.1016/j.comnet.2026.112226 (2026).

[84] Por, L. Y. et al. A Systematic Literature Review on AI-Based Methods and Challenges in Detecting Zero-Day Attacks. *IEEE Access.* **12**, 144150–144163. 10.1109/ACCESS.2024.3455410 (2024).

[85] Reddy, G. R., Kollu, B. S. K., Elshafie, V. N., Qamar, S. & H., & A novel blockchain-federated learning framework with quantum neural networks and wavelet transforms for secure IoT healthcare monitoring. *Biomed. Signal Process. Control*. **113**, 108759. 10.1016/j.bspc.2025.108759 (2026).

[86] Shuaib, M. Federated deep learning for secure and energy-efficient cyber threat mitigation in smart grid automation. *Sustainable Computing: Inf. Syst.* **48**, 101248. 10.1016/j.suscom.2025.101248 (2025).

[87] Wahab, F., Ma, S., Zhao, Y. & Shah, A. An explainable three-way neural network approach for intrusion detection in IoT ecosystem. *Internet Things*. **33**, 101722. 10.1016/j.iot.2025.101722 (2025).

[88] Umar, M. A., Chen, Z., Shuaib, K. & Liu, Y. Effects of feature selection and normalization on network intrusion detection. *Data Sci. Manage.* **8** (1), 23–39. 10.1016/j.dsm.2024.08.001 (2025).

[89] Katbi, A. & Ksantini, R. One-class IoT anomaly detection system using an improved interpolated deep SVDD autoencoder with adversarial regularizer. *Digit. Signal Proc.* **162**, 105153. 10.1016/j.dsp.2025.105153 (2025).

[90] Raza, M. M. et al. Malware detection and AI integration: A systematic review of current trends and future directions. *Comput. Model. Eng. Sci.* **146** (3). http://www.techscience.com/CMES/v146n3/66787 (2026).

[91] Wahab, F. et al. A ranked filter-based three-way clustering strategy for intrusion detection in highly secure IoT networks. *Comput. Electr. Eng.* **127**, 110514. 10.1016/j.compeleceng.2025.110514 (2025).

[92] Faramondi, L., Flammini, F., Guarino, S. & Setola, R. A hybrid behavior- and Bayesian network-based framework for cyber–physical anomaly detection. *Comput. Electr. Eng.* **112**, 108988. 10.1016/j.compeleceng.2023.108988 (2023).

[93] Kasina, D., Reddy, V. S., Rajamanickam, S., Jose, J. & Palani, B. A novel intrusion detection system for class imbalance datasets using hybrid sampling with deep learning techniques. *Inf. Sci.* **741**, 123208. 10.1016/j.ins.2026.123208 (2026).

[94] Wang, J. et al. Self-learning model fusion for network anomaly detection: A hybrid CNN-LSTM-transformer framework. *PLOS ONE*. **20** (10), e0332502. 10.1371/journal.pone.0332502 (2025). 41160632 10.1371/journal.pone.0332502 PMC12571298

[95] Alansary, S. A., Ayyad, S. M., Talaat, F. M. & Saafan, M. M. Emerging AI threats in cybercrime: a review of zero-day attacks via machine, deep, and federated learning. *Knowl. Inf. Syst.* **67** (11), 10951–10987. 10.1007/s10115-025-02556-6 (2025).

[96] Li, J., Ersotelos, N., Tsompanas, M. A. & Epiphaniou, G. Challenges and opportunities of synthetic data generation for machine learning-based intrusion detection systems in in-vehicle networks. *Veh. Commun.* **58**, 100998. 10.1016/j.vehcom.2025.100998 (2026).

[97] Makhadmeh, S. N. et al. A crossover-integrated Marine Predator Algorithm for feature selection in intrusion detection systems within IoT environments. *Internet Things*. **31**, 101536. 10.1016/j.iot.2025.101536 (2025).

[98] Alzaylaee, M. K., Almarshad, F. A., Gashgari, G. A., Algawiaz, D. & Alzahrani, A. I. A. Advancing cybersecurity: AI-driven computer vision and machine learning models for real-time threat detection and prevention. *J. Eng. Res.*10.1016/j.jer.2026.01.014 (2026).

[99] Rezaei, H., Taheri, R. & Shojafar, M. FedLLMGuard: A federated large language model for anomaly detection in 5G networks. *Comput. Netw.* **269**, 111473. 10.1016/j.comnet.2025.111473 (2025).

[100] Gaber, M., Ahmed, M. & Janicke, H. Zero day malware detection with Alpha: Fast DBI with Transformer models for real world application. *Comput. Electr. Eng.* **128**, 110751. 10.1016/j.compeleceng.2025.110751 (2025).

[101] Guo, Y. A review of Machine Learning-based zero-day attack detection: Challenges and future directions. *Comput. Commun.* **198**, 175–185. 10.1016/j.comcom.2022.11.001 (2023). 10.1016/j.comcom.2022.11.001 PMC9890381 36741076

[102] Wani, A. et al. AI-driven botnet detection in IoT networks: A comprehensive research review. *Comput. Sci. Rev.* **61**, 100941. 10.1016/j.cosrev.2026.100941 (2026).

[103] Wu, C. et al. Robust Network Intrusion Detection via Temporal Contrastive Graph Learning. *IEEE Trans. Inf. Forensics Secur.* **20**, 1475–1486. 10.1109/tifs.2025.3530702 (2025).

[104] Yang, J. et al. LLM-AE-MP: Web Attack Detection Using a Large Language Model with Autoencoder and Multilayer Perceptron. *Expert Syst. Appl.* **274**, 126982. 10.1016/j.eswa.2025.126982 (2025).

[105] Dai, Z. et al. An intrusion detection model to detect zero-day attacks in unseen data using machine learning. *PLOS ONE*. **19** (9), e0308469. 10.1371/journal.pone.0308469 (2024). 39259729 10.1371/journal.pone.0308469 PMC11389943

[106] Caro-Martínez, M. et al. iSee: A case-based reasoning platform for the design of explanation experiences. *Knowl. Based Syst.* **302**, 112305. 10.1016/j.knosys.2024.112305 (2024).

[107] Rodriguez, K. S. R. et al. Secure Development Methodology for Full Stack Web Applications: Proof of the Methodology Applied to Vue.js, Spring Boot and MySQL. *Computers Mater. Continua*. **85** (1), 1807–1858. 10.32604/cmc.2025.067127 (2025).

[108] Canavese, D., Ferreira, A., Kamm, L. & Quesada Rodriguez, A. Uncovering challenges of cybersecurity cross-regulation in EU legislation. *Inf. Softw. Technol.* **193**, 108033. 10.1016/j.infsof.2026.108033 (2026).

[109] Russo, D., Miori, V., Tolomei, G. & Belli, D. Home automation interoperability: two decades of lessons learned and future prospects into the development of IoT ecosystems. *Internet Things*. **37**, 101906. 10.1016/j.iot.2026.101906 (2026).

[110] Conrad, E., Misenar, S. & Feldman, J. Chapter 8 - Domain 7: Security Operations. In E. Conrad, S. Misenar, & J. Feldman (Eds.), CISSP^®^ Study Guide (Fourth Edition) (pp. 361–457). Syngress. 10.1016/B978-0-443-18734-6.00006-4 (2023).

[111] Shyaa, M. A. et al. Evolving cybersecurity frontiers: A comprehensive survey on concept drift and feature dynamics aware machine and deep learning in intrusion detection systems. *Eng. Appl. Artif. Intell.* **137**, 109143. 10.1016/j.engappai.2024.109143 (2024).

[112] Villegas-Ch, W., Gutierrez, R., Sánchez-Salazar, I. & Mera-Navarrete, A. Adaptive Security Framework for the Internet of Things: Improving Threat Detection and Energy Optimization in Distributed Environments. *IEEE Access.* **12**, 157924–157944. 10.1109/ACCESS.2024.3486983 (2024).

[113] Adenusi, D. A., Oladimeji, O. O., Oyekola, T. A. & Olagunju, K. S. Data-driven network intrusion detection using optimized machine learning algorithms. *Frankl. Open.* **12**, 100339. 10.1016/j.fraope.2025.100339 (2025).

[114] Jeniffer, J. T., S, R. & A, C., & Efficient intrusion detection in wireless sensor networks using MHCEGRU with cross-layer monitoring and cryptographic security. *Knowl. Based Syst.* **332**, 114707. 10.1016/j.knosys.2025.114707 (2026).

[115] Khalid, A., Zainal, A., Ghaleb, F. A., Al-rimy, B. A. S. & Ahmed, Y. A hybrid feature selection method for advanced persistent threat detection. *Comput. Mater. Continua*, **84**(3), 5665–5691. 10.32604/cmc.2025.063451 (2025).

[116] Liu, P., Zhao, Y. & Feng, Y. PER-AE-DRL: A malicious traffic detection model based on prioritized experience replay and adversarial mechanism. *J. Inform. Secur. Appl.* **96**, 104298. 10.1016/j.jisa.2025.104298 (2026).

[117] Singha, M. F., Patgiri, R., Shamsi, Z. & Singh, L. D. Zero-day attack detection with a Dynamic-Weighted Contractive Autoencoder and GAN-based evaluation. *Comput. Electr. Eng.* **128**, 110650. 10.1016/j.compeleceng.2025.110650 (2025).

[118] Xu, C. et al. Few-shot network intrusion detection method based on multi-domain fusion and cross-attention. *PLOS ONE*. **20** (7), e0327161. 10.1371/journal.pone.0327161 (2025). 40601763 10.1371/journal.pone.0327161 PMC12221178

